# Multiomics Analysis Identifies SOCS1 as Restraining T Cell Activation and Preventing Graft‐Versus‐Host Disease

**DOI:** 10.1002/advs.202200978

**Published:** 2022-05-18

**Authors:** Huidong Guo, Ruifeng Li, Ming Wang, Yingping Hou, Shuoshuo Liu, Ting Peng, Xiang‐Yu Zhao, Liming Lu, Yali Han, Yiming Shao, Ying‐Jun Chang, Cheng Li, Xiao‐Jun Huang

**Affiliations:** ^1^ Peking University Institute of Hematology National Clinical Research Center for Hematologic Disease Beijing Key Laboratory of Hematopoietic Stem Cell Transplantation School of Life Sciences Peking University People's Hospital Peking University Beijing 100044 China; ^2^ Peking‐Tsinghua Center for Life Sciences Peking University Beijing 100080 China; ^3^ Institute for Immunology and School of Medicine Tsinghua University Beijing 100084 China; ^4^ Beijing Tsinghua Changgeng Hospital Beijing 102218 China; ^5^ Shanghai Institute of Immunology Shanghai Jiaotong University School of Medicine 280 South Chongqing Road Shanghai 200025 China; ^6^ Shanghai Jiayin Biotechnology, Ltd. Shanghai 200092 China; ^7^ Center for Statistical Science Center for Bioinformatics Peking University Beijing China; ^8^ Research Unit of Key Technique for Diagnosis and Treatments of Hematologic Malignancies (2019RU029) Chinese Academy of Medical Sciences Beijing 100730 China

**Keywords:** graft‐versus‐host disease (GVHD), hematopoietic stem cell transplantation (HSCT), multiomics, SOCS1, T cell tolerance

## Abstract

Graft‐versus‐host disease (GVHD) is a major life‐threatening complication of allogeneic hematopoietic stem cell transplantation (allo‐HSCT). Inflammatory signaling pathways promote T‐cell activation and are involved in the pathogenesis of GVHD. Suppressor of cytokine signaling 1 (*SOCS1*) is a critical negative regulator for several inflammatory cytokines. However, its regulatory role in T‐cell activation and GVHD has not been elucidated. Multiomics analysis of the transcriptome and chromatin structure of granulocyte‐colony‐stimulating‐factor (G‐CSF)‐administered hyporesponsive T cells from healthy donors reveal that G‐CSF upregulates *SOCS1* by reorganizing the chromatin structure around the *SOCS1* locus. Parallel in vitro and in vivo analyses demonstrate that *SOCS1* is critical for restraining T cell activation. Loss of *Socs1* in T cells exacerbates GVHD pathogenesis and diminishes the protective role of G‐CSF in GVHD mouse models. Further analysis shows that SOCS1 inhibits T cell activation not only by inhibiting the colony‐stimulating‐factor 3 receptor (CSF3R)/Janus kinase 2 (JAK2*)*/signal transducer and activator of transcription 3 (STAT3*)* pathway, but also by restraining activation of the inflammasome signaling pathway. Moreover, high expression of *SOCS1* in T cells from patients correlates with low acute GVHD occurrence after HSCT. Overall, these findings identify that SOCS1 is critical for inhibiting T cell activation and represents a potential target for the attenuation of GVHD.

## Graphical abstract

Granulocyte‐colony‐stimulating‐factor (G‐CSF) activates signal transducer and activator of transcription 3 (STAT3) by the colony‐stimulating‐factor 3 receptor (CSF3R), then phosphorylated STAT3 enters into nucleus and upregulates suppressor of cytokine signaling 1 (*SOCS1*) by reorganizing chromatin structure around the *SOCS1* locus. Activating SOCS1 prevents T‐cell activationand graft‐versus‐host disease by inhibiting CSF3R, STAT3, and NOD‐, LRR‐ and pyrin domain‐containing protein 3 (NLRP3) inflammasome activation.

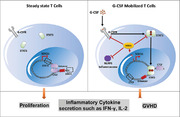

## 1 Introduction

Allogeneic hematopoietic stem cell transplantation (allo‐HSCT) remains a curative therapy for hematological malignancies.^[^ ^1^ ^]^ However, graft‐versus‐host disease (GVHD), a major cause of morbidity and mortality after HSCT, remains an obstacle to the success of transplantation and threatens the survival of patients after HSCT.^[^ ^2^ ^]^ Among all patients undergoing allo‐HSCT, 30–50% have acute GVHD (aGVHD; grade I to IV), and 14% have severe aGVHD (grade III to IV).^[^ ^3^ ^]^ Donor T cells that destroy recipient tissues are the main mediators of GVHD.^[^ ^4^ ^]^ Proinflammatory cytokines such as interferon‐*γ* (IFN‐γ) and interleukin‐6 (IL‐6) secreted by alloreactive donor T cells are involved in the pathogenesis of GVHD, inducing direct cytotoxic effects on host tissues, activation of immune effector cells, and differentiation of proinflammatory T helper 1 (Th1) and T helper 17 (Th17) populations.^[^ ^5^ ^]^ Therefore, targeting key signal transduction molecules in the form of proinflammatory signaling activating the T cell pathway has become an alternative approach for GVHD prevention and treatment.

The Janus kinase (JAK) and signal transducer and activator of transcription (STAT) pathway is one of the most important inflammatory signaling pathway that regulates T cell activation in the pathogenesis of GVHD.^[^ ^6^ ^]^ For example, IL‐6 activates JAK2 and leads to downstream phosphorylation of STAT3, which contributes to GVHD severity in experimental systems.^[^ ^5^, ^7^ ^]^ Tocilizumab, which targets the IL‐6 receptor and inhibits the JAK2/STAT3 signaling pathway, has been approved for the treatment of rheumatoid arthritis^[^ ^8^ ^]^ and was found to ameliorate human GVHD in several studies.^[^ ^9^ ^]^ Moreover, other inhibitors directly targeting JAK/STAT signaling, such as ruxolitinib, tofacitinib, and itacitinib, have been reported to mitigate GVHD in murine models.^[^ ^6^, ^10^ ^]^ NOD‐, LRR‐ and pyrin domain‐containing protein 3 (NLRP3) inflammasome is another inflammatory signaling, which is activated by dual signals and produces activated IL‐1β to participate in a variety of inflammatory immune response.^[^ ^11^ ^]^ Previous study showed that in the GVHD mouse models, allogenic T cells differentiated into Th17 cells in response to IL‐1β in an *NLRP3* inflammasome dependent manner.^[^ ^12^ ^]^

The suppressor of cytokine signaling (SOCS) family is a downstream target of the JAK/STAT pathway, inhibiting JAK/STAT phosphorylation and activation via a negative feedback loop.^[^ ^13^ ^]^ SOCS1, as the most potent member of the SOCS family, negatively regulates the JAK/STAT pathway and is essential for inhibiting secretion of the proinflammatory cytokine IFN‐γ.^[^ ^14^ ^]^ *Socs1^−/−^* mice die at 2–3 weeks of age from inflammation and can be rescued by genetic deletion of IFN‐γ.^[^ ^15^ ^]^ SOCS1 deficiency promotes dysregulated JAK/STAT signaling related to a number of immune disorders, including systemic lupus erythematosus, scleritis, and asthma.^[^ ^16^ ^]^ By affecting the kinase inhibitory region (KIR) domain of SOCS1, which can directly inhibit JAK kinase activity, SOCS1 mimetics were found to inhibit experimental autoimmune encephalomyelitis (EAE) and suppressed T cell activation and IL‐17A production.^[^ ^17^ ^]^ These data strongly indicate that SOCS1 acts as a negative regulator of JAK/STAT signaling and inhibits the inflammatory response in T cells. However, the regulatory role of SOCS1 in GVHD has not been elucidated.

Granulocyte colony‐stimulating factor (G‐CSF) has been widely used in HSCT and accepted as an immune regulator that affects both innate immune cells and adaptive immune cells, especially T cells.^[^ ^18^ ^]^ Several studies have proven that G‐CSF can induce T cell hyporesponsiveness by decreasing T cell proliferation and IL‐2 production, skewing T cell differentiation into Th2 populations in healthy HSCT donors.^[^ ^19^ ^]^ Moreover, murine GVHD models demonstrated that donor pretreatment with G‐CSF hampered GVHD.^[^ ^20^ ^]^ Therefore, investigating the intrinsic mechanism by which G‐CSF regulates T cell tolerance could provide a new avenue for the discovery of T cell tolerance regulators. Recent advances in chromatin structure analytic technologies, including transposase‐accessible chromatin with high‐throughput sequencing (ATAC‐seq) and genome‐wide chromosome conformation capture (Hi‐C) assays,^[^ ^21^ ^]^ have aided the delineation of the spatial regulation network of transcription factors regulating gene expression. By employing these technologies, we can explore the underlying molecular mechanism by which G‐CSF induces T cell tolerance and discover new immune tolerance regulators.

In this study, we systematically analyzed the dynamics of the T cell transcriptome and 3D genome in contexts ranging from steady state to a G‐CSF‐administered hyporesponsive state and found that the *SOCS1* expression level was upregulated via G‐CSF reorganization of the chromatin structure of the *SOCS1* locus. Parallel in vitro and in vivo analyses demonstrated that SOCS1 inhibited T cell activation and dampened GVHD by inhibiting several inflammatory signaling pathway including JAK2/STAT3 signaling and NLRP3 inflammasome signaling. Moreover, high expression of *SOCS1* in T cells from patients correlated with low aGVHD occurrence after HSCT. Overall, our investigation identified the critical role of SOCS1 in controlling GVHD and a new avenue for therapeutic interventions to attenuate GVHD.

## 2 Results

### 2.1 Multiomics Analysis Identified the Transcriptome and Epigenome Profile of Hyporesponsive T Cells from Healthy Donors Administrated with G‐CSF

**Figure 1:**
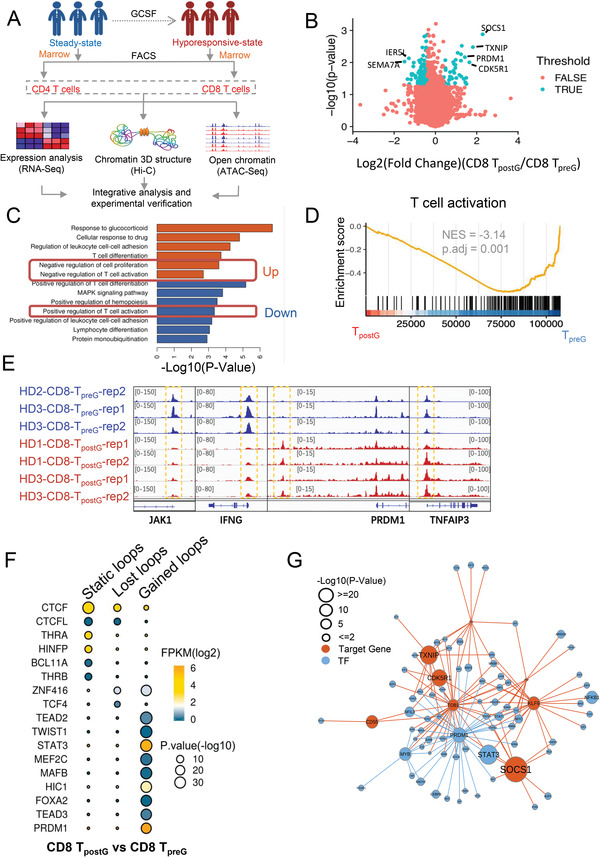
Multiomics‐analyzed transcriptome and 3D genome of steady state T cells and G‐CSF‐administrated hyporesponsive T cells. A) Outline of the experiments and analyses in this study. We performed in situ Hi‐C, RNA‐seq, and ATAC‐seq experiments on paired T cells (CD4 and CD8) from three healthy donors before and after G‐CSF mobilization in vivo. B) Volcano plot comparing CD8 T_preG_ and CD8 T_postG_. The *X*‐axis shows the fold change (log2). Among the genes, 54 genes were significantly upregulated, and 78 genes were significantly downregulated. C) Gene pathway enrichment analysis for the upregulated (red) and downregulated (blue) genes in CD8 T_postG_ cells. D) T‐cell‐activation‐associated chromatin accessibility changes in CD8 T_preG_ and T_postG_. The T‐cell‐activation‐associated ATAC‐seq data base on Bediaga et al.^[^ ^44^ ^]^ E) The UCSC (University of California, Santa Cruz) browser views showing ATAC‐seq of representative open chromatin accessibility genes (*PRDM1* and *TNFAIP3*) and close chromatin accessibility genes (*JAK1* and *IFNG*) in CD8 T_postG_ cells compared with CD8 T_preG_ cells. F) Motif results predicted by HOMER (Hypergeometric Optimization of Motif EnRichment) software to have different types of loops in CD8 T_postG_ cells. CTCF is the transcription factor most significantly enriched in static loops. This finding is consistent with the existing literature. G) The regulatory network map of highly expressed genes and enhanced transcription factors in CD8 T_preG_ and CD8 T_postG_ cells. Red dots represent transcription factors, and purple dots represent target genes. The regulatory relationships between transcription factors and genes are based on Yan et al.^[^ ^22^ ^]^

To investigate the dynamics of the transcriptome and epigenome in T cells in states ranging from steady state to a hyporesponsive state, we assessed the transcriptome, chromatin accessibility, and 3D genome landscape of steady state cluster of differentiation (CD) 4^+^ and CD8^+^ T cells (CD4 T_preG_ and CD8 T_preG_, respectively) and G‐CSF‐administered CD4^+^ and CD8^+^ T cells (CD4 T_postG_ and CD8 T_postG_, respectively) in human bone marrow (BM) samples (**Figure** **1**A). The increases observed in a core set of genes, including suppressor of cytokine signaling 1 (*SOCS1*) and *PR*/*SET* domain 1 (*PRDM1*), were similar in CD4 T_postG_ and CD8 T_postG_ cells (Figure 1B and Figure S1A–C (Supporting Information)). Both CD4 T_postG_ and CD8 T_postG_ cells showed a significant upregulation of genes related to negative regulation of cell proliferation (Figure 1C and Figure S1D (Supporting Information)). The ATAC‐seq data showed that there was a negative correlation of T cell activation with peak enrichment (Ref.44) in CD8 T_postG_ cells (Figure 1D and Figure S2A (Supporting Information)). The T‐cell‐activation‐related gene *JAK1* and the proinflammatory gene *IFNG* showed decreased accessibility in CD8 T_postG_ cells, while the T‐cell‐tolerance‐regulated gene *PRDM1* and the tumor necrosis factor alpha‐induced protein 3 (*TNFAIP3*) gene (a gene negatively regulating NF‐κB signaling) showed increased accessibility in CD8 T_postG_ cells (Figure 1E). We also found that T‐cell‐activation‐related transcription factors (TFs), such as JunB proto‐oncogene (JUNB), activator protein 1 (AP‐1), and Fos‐related antigens 1 (FRA1), were suppressed in both CD8 T_postG_ and CD4 T_postG_ cells compared with CD8 T_preG_ and CD4 T_preG_ cells (Figure S2B–E, Supporting Information). From Hi‐C data, we obtained high‐resolution maps (5 kb) of the 3D genome structure, including A/B compartments, topologically associating domain (TAD) structures, and loop structures, of all the samples (Figure S3A–H, Supporting Information). More than 53% of the genes upregulated in CD8 T_postG_ cells compared with CD8 T_preG_ cells, including *SOCS1*, *PRDM1*, and kruppel like factor 9 (*KLF9*), were found to be located in loop anchor regions (Figure S4A, Supporting Information, *p* < 1 × 10^−16^). There were distinct chromatin loop structures in both the pre and post G‐CSF administrated CD4^+^ T cells and CD8^+^ T cells (Figure S4B,C, Supporting Information). Compared with CD8 T_preG_ cells, CD8 T_postG_ cells showed enrichment of STAT3 and PRDM1 motifs in the gained loop anchors and high *STAT3* and *PRDM1* expression (Figure 1F), which is consistent with the ATAC‐seq results (Figure 1E and Figure S2B (Supporting Information)). This finding suggests that STAT3 may be a structural protein that mediates the gain of chromatin loops. According to the relationship between TFs and target genes,^[^ ^22^ ^]^ we constructed the network of upregulated genes and enhanced regulatory transcription factors in CD8^+^ cells, which showed that *SOCS1* was highly expressed and that STAT3 was the TF that most strongly regulated *SOCS1* (Figure 1G). These results indicated that T cells from healthy donors administrated with G‐CSF showed hyporesponsive transcriptome and epigenome profiling.

### 2.2 G‐CSF Upregulated *SOCS1* by Reorganizing the Chromatin Structure

**Figure 2:**
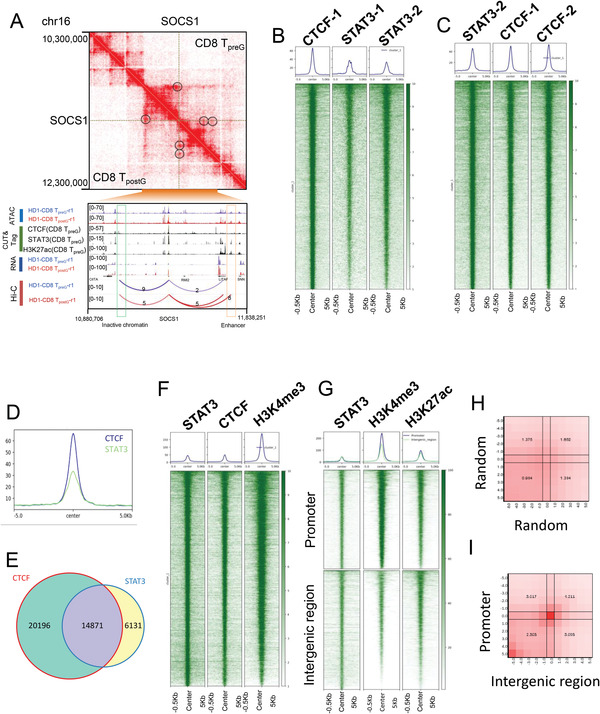
G‐CSF upregulated *SOCS1* expression level by STAT3‐mediated chromatin structure reorganization. A) Top: Hi‐C interaction matrix of a region (*chr16*: 10.3–12.3 Mb) in CD8 T_preG_ cells around the *SOCS1* gene. Bottom: Genome browser view of CTCF and STAT3 binding sites, histone modifications, chromatin accessibility, gene expression chromatin states, and 3D genome interactions around the *SOCS1* gene in CD8 T cells. The green box represents the region of chromatin with reduced interactions with *SOCS1* after G‐CSF mobilization. The yellow box represents the region of chromatin with increase interactions with *SOCS1* after G‐CSF mobilization. B) Heatmaps displaying whole‐genome STAT3 and CTCF colocalization in CD8 T_preG_ cells according to CUT&Tag data (the top 5000 CTCF peaks in CD8 T_preG_ cells). C) Heatmaps displaying whole‐genome STAT3 and CTCF colocalization in CD8 T_preG_ cells according to CUT&Tag data (the top 5000 STAT3 peaks in CD8 T_preG_ cells). D) Aggregate plot of CTCF binding (blue line) and STAT3 binding (green line) at ±5.0 kb from the CTCF peaks in CD8 cells. E) Venn diagram showing the overlap between CTCF peaks (red) and STAT3 peaks (blue) in CD8 T_preG_ cells. *p* < 1 × 10^−10^, hypergeometric test. F) Heatmaps displaying STAT3 occupancy and active promoters (the top 5000 STAT3 peaks in CD8 T_preG_ cells). G) The peaks of STAT3 binding are classified as belonging to two clusters. The first cluster is the promoter region, which overlaps with the promoters of all genes (±1 kb around the transcription start site), and the second represents intergenic regions. H) Heatmap of the interaction between the promoter region and the intergenic region in the spatial interaction between enhancers and promoters. I) A random selection of the same number of enhancers and promoter peaks has no significant spatial interaction.

We next explored the regions of spatial interaction with the promoter of *SOCS1* and determined which transcription factors bind to these regions. On chromosome 16, the *SOCS1* gene was located within one TAD in CD8 T_preG_ cells (**Figure** **2**A). We investigated the chromatin spatial structure, histone modification, and TF binding sites around the *SOCS1* gene. The genome browser view of CTCF and STAT3 binding sites suggested that the CTCF protein mediates the interaction between the *SOCS1* locus and the downstream chromatin region and that STAT3 proteins mediate the interaction between *SOCS1* and upstream enhancer. From Hi‐C data, the interaction between the *SOCS1* locus and downstream inactive chromatin was found to be weakened and the interaction between *SOCS1* and upstream enhancer was found to be enhanced in CD8 T_postG_ cells compared to CD8 T_preG_ cells (Figure 2A). These results suggest that a new association of STAT3 with *SOCS1* expression emerged during G‐CSF‐induced T cell hyporesponsiveness. In support of this hypothesis, genome‐wide statistics showed that genes with long‐range interactions with inactive chromatin tended to be expressed at low levels, while genes with long‐range interactions with enhancers tended to be highly expressed (Figure S4D, Supporting Information). To investigate whether STAT3 competes with CTCF in regulating target genes, we performed chromatin immunoprecipitation with sequencing (ChIP‐seq) and cleavage under targets and tagmentation (CUT&Tag) experiments to detect the colocalization of STAT3 and CTCF. For example, many of the binding sites of STAT3 and CTCF are colocalized in and around *SOCS1* (Figure S5A, Supporting Information) and thioredoxin interacting protein (*TXNIP*) (Figure S5B, Supporting Information), which are upregulated after G‐CSF mobilization. Furthermore, STAT3 and CTCF colocalized in the whole genome analysis of human CD8 T cells and GM12878 cell lines (Figure 2B–D and Figure S5C–F (Supporting Information)). There was a significant overlap between the CTCF peaks and the STAT3 peaks in CD8 T cells, as shown by Venn diagram (*p* < 1 × 10^−10^, Figure 2E). The peaks of STAT3 binding are enriched in promoter and enhancer regions (Figure 2F,G). Then, we classified the peaks of STAT3 binding into promoter regions or enhancer regions (Figure 2G and Figure S5G,H (Supporting Information)), and there is a significant spatial interaction between the promoter regions and enhancer regions (Figure 2H,I). These results strongly suggest that the STAT3 complex is involved in enhancer and promoter interactions. Consistent with our observation, previous studies showed STAT3 could regulate chromatin topology and mediate transcription during T cell differentiation.^[^ ^23^ ^]^ STAT4 binding in the genome contributes to the specification of the nuclear architecture around *IFNG* during Th1 differentiation.^[^ ^24^ ^]^ Furthermore, we observed both CTCF and STAT3 foci in the nuclei of Jurkat cells by immunofluorescence staining (Figure S5J, Supporting Information). Collectively, these results suggest that a STAT3‐mediated enhancer–promoter interaction might induce *SOCS1* expression during the in vivo induction of T cell hyporesponsiveness.

Furthermore, we validated the direct upregulation of *SOCS1* expression induced by G‐CSF in purified CD3^+^ T cells from 7 independent heathy donor bone marrow samples in vitro. The results showed that G‐CSF stimulation led to a peak in *SOCS1* messenger RNA (mRNA) production after 4 h of culture, followed by upregulation after 72 h of culture (Figure S6A–C, Supporting Information). After culturing CD3^+^ T cells for 72 h with G‐CSF stimulation in vitro, the receptor of G‐CSF, colony‐stimulating‐factor 3 receptor (CSF3R), expression level was significantly increased (Figure S6D,E, Supporting Information). Consistent with previous studies,^[^ ^19c^ ^]^ IL‐2 was decreased in the G‐CSF treatment group (Figure S6F,G, Supporting Information), and T cell activation marker such as CD25 and CD69 showed no difference between phosphate‐buffered saline (PBS) treatment group and G‐CSF treatment group (Figure S6H,I, Supporting Information). Moreover, we investigated phosphorylation level of STAT family proteins including P‐STAT1, P‐STAT3, and P‐STAT5 in G‐CSF‐treated T cells and found that only phosphorylation of STAT3 was elevated in both G‐CSF‐treated CD4^+^ and CD8^+^ T cells (Figure S6J,K, Supporting Information). These results indicated that G‐CSF could directly upregulate *SOCS1* expression level and activated STAT3 phosphorylation in T cells in vitro.

### 2.3 Elevating *SOCS1* in Human Primary T Cells Induced T Cell Hyporesponsiveness

**Figure 3:**
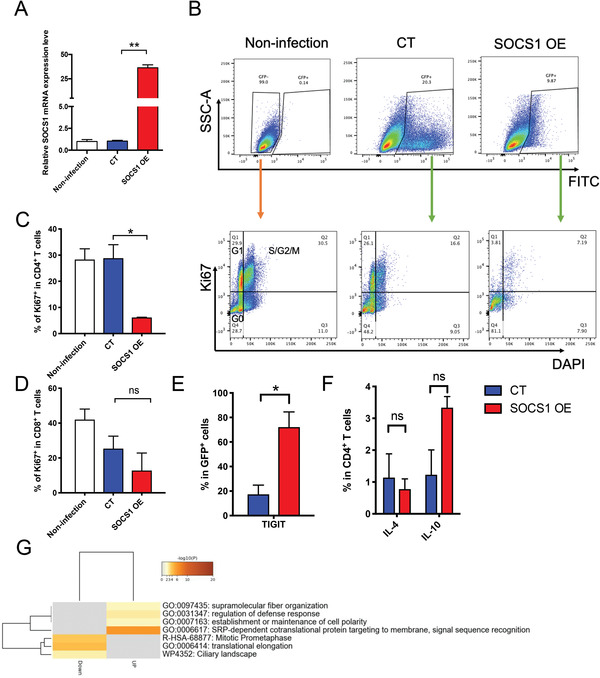
Highly expressed *SOCS1* in human primary T cells inhibited T cell activation. A) *SOCS1* was overexpressed by lentivirus in CD3^+^ T cells from healthy donor bone marrow. Quantitative real‐time (RT)‐PCR was used to detect *SOCS1* expression levels. B) Flow cytometric analysis of proliferation in GFP^+^ cells. C,D) Percentage of Ki67^+^ cells in CD4^+^ cells (C) or CD8^+^ cells (D). E) The expression level of TIGIT detected by flow cytometry. F) IL‐4 and IL‐10 secretion levels in GFP^+^CD4^+^ T cells. G) Gene pathway enrichment analysis for the upregulated genes and downregulated genes in SOCS1‐overexpressing T cells compared with control T cells. Error bars represent the mean ± SEM values from 3 independent experiments, *p < 0.05, **p < 0.01.

To investigate the role of SOCS1 in maintaining T cell tolerance, we used lentivirus to overexpress *SOCS1* in steady‐state T cells and found that the *SOCS1* expression level was increased ≈30‐fold in the SOCS1 overexpression (SOCS1 OE) group compared with the control (CT) group (**Figure** **3**A). High expression of *SOCS1* inhibited T cell proliferation, and more T cells were blocked in the G0 stage in the SOCS1 OE group than in the CT or noninfection group (Figure 3B–D and Figure S7A (Supporting Information)). Moreover, high *SOCS1* expression in T cells also promoted T cell immunoreceptor with Ig and ITIM domains (TIGIT) expression (Figure 3E and Figure S7B,C (Supporting Information)). There were no significant differences in the secretion of cytokines, such as IFN‐γ, IL‐2, IL‐17, IL‐4, and IL‐10, by CD4^+^ T and CD8^+^ T cells between the SOCS1 OE group and CT group (Figure 3F and Figure S8A–C (Supporting Information)). Gene ontology (GO) analysis showed that the mitotic prometaphase signaling pathway was enriched in genes downregulated in the SOCS1 OE group compared with the CT group, which is consistent with the SOCS1 OE T cells that were arrested in the G0 stage (Figure 3G). We further purified CD3^+^ T cells from G‐CSF‐treated healthy donors and knocked down *SOCS1* expression by small interfering RNA (siRNA). Of the three siRNA sequences tested, two (siRNA‐2 and siRNA‐3) were found to be effective in reducing *SOCS1* mRNA expression, as assessed by quantitative reverse transcriptase‐polymerase chain reaction (qRT‐PCR) (Figure S9A, Supporting Information). The group with decreased *SOCS1* expression had lower levels of IL‐10 secretion, as evidenced by FACS (fluorescence‐activated cell sorting) and ELISA (enzyme‐linked immunosorbent assay), than the NC (negative control) group, but there was no statistically significant difference (Figure S9B–D, Supporting Information). This lack of significance could be explained by the fact that *SOCS1* expression was not completely knocked down by siRNA in vitro; therefore, in vivo experiments with mouse models with *Socs1*‐specific knockout in T cells are needed.

### 2.4 Ablation of *Socs1* in T Cells Exacerbated Mouse GVHD

**Figure 4:**
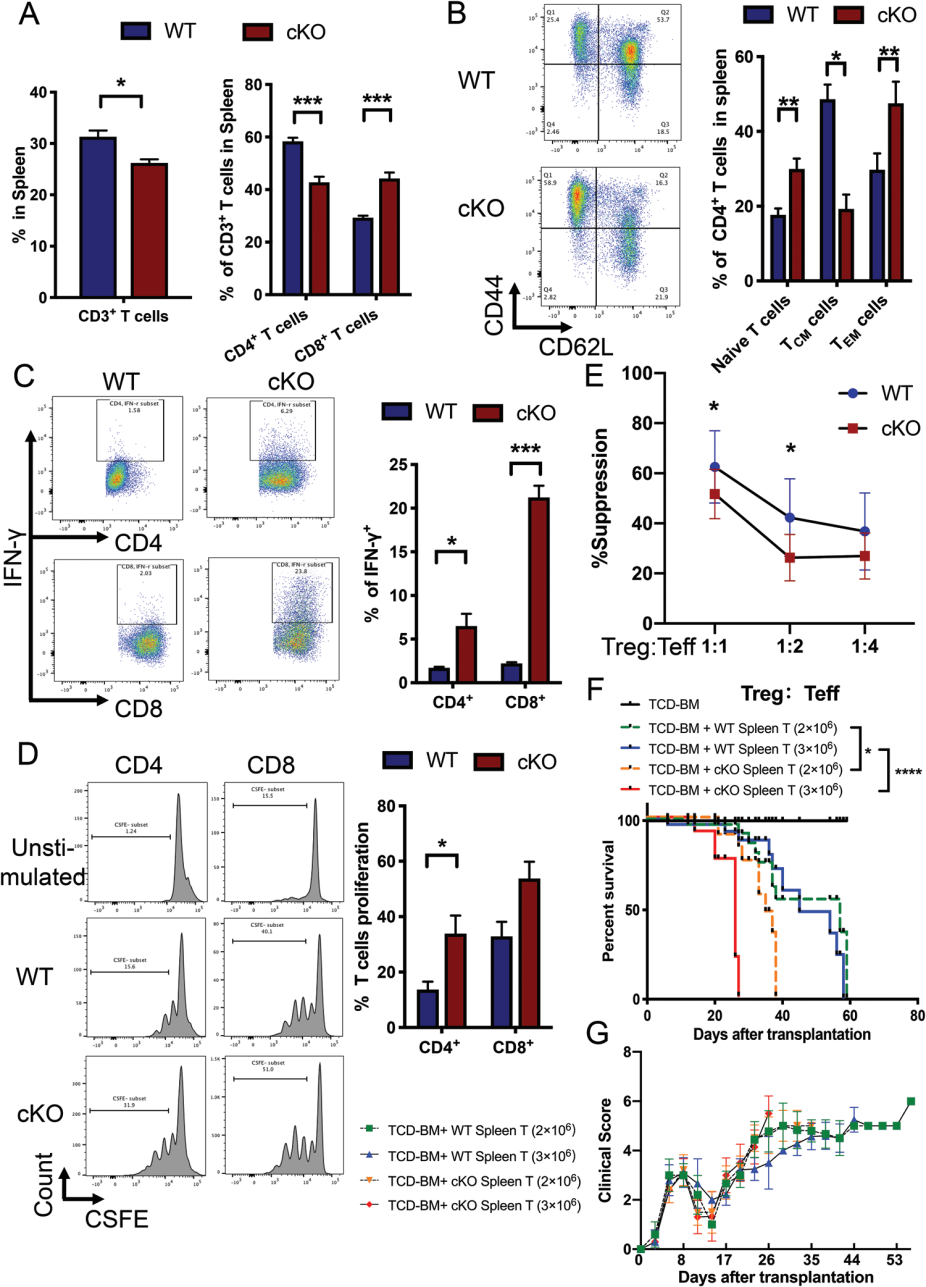
*Socs1* deficiency in T cells exacerbated mice GVHD. A) Percentage of CD3^+^ T cells in the spleen from WT or cKO mice (left). Percentage of CD4^+^ and CD8^+^ T cells in CD3^+^ T cells in the spleen from WT or cKO mice (right). B) Representative flow cytometry results and percentages show the CD62L and CD44 expression levels on CD4^+^ T cells from the spleens of WT or cKO mice. Naïve T cells: CD62L^+^CD44^−^; central memory T cells (T_CM_): CD62L^+^CD44^+^; effector memory T cells (T_EM_): CD62L^−^CD44^+^. C) Representative flow cytometry results and percentages show the *IFN‐γ* secretion level of CD4^+^ and CD8^+^ T cells from the spleens of WT or cKO mice. D) CFSE analysis showed the proliferation ability of T cells from the spleens of WT or cKO mice. E) Treg cells (CD4^+^CD25^+^) from the spleens of WT or cKO mice were cocultured with Teff cells (CD4^+^CD25^−^) from the spleens of WT mice. The proliferation ability of Teff cells was detected by CFSE. F) Survival curves of GVHD mouse models. A total of 5 × 10^6^ TCD‐BM from WT mice and 3 × 10^6^ T cells from the spleens of the WT or cKO donor mice were transplanted into the corresponding recipient mice (blue vs red). A total of 5 × 10^6^ TCD‐BM from WT mice and 2 × 10^6^ T cells from the spleens of the WT or cKO donor mice were transplanted into the corresponding recipient mice (green vs orange). 10 mice per group. G) Clinical scores of GVHD mouse models. The experiment was repeated at least 3 times, with 4–6 mice per group. Error bars represent the mean ± SEM, ***p < 0.001, **p < 0.01, *p < 0.05.

To further investigate the essential role of SOCS1 in maintaining T cell tolerance, we established a T cell‐specific *Socs1* conditional knockout (cKO; *LckCre*‐*Socs1^fl/fl^*) mouse model. Consistent with previous studies,^[^ ^25^ ^]^ most cKO mice survived longer than 6 months. Occasionally, cKO mice developed dermatitis at 4 weeks (3 in 50 mice, Figure S10A, Supporting Information). There was also splenomegaly in the cKO mice, which was not apparent in the *Socs1^fl/fl^* (wild type (WT)) mice (Figure S10B, Supporting Information). Flow cytometry analysis showed that CD3^+^ T cells were decreased in cKO mice compared with WT mice (**Figure** **4**A). The transposition of the CD4/CD8 ratio in cKO mice represented a decrease in CD4^+^ T cells and an increase in CD8^+^ T cells compared with the respective levels in WT mice (Figure 4A). The ratio of naïve T cells and effector memory T cells (T_EM_) cells were increased and central memory T cells (T_CM_) was decreased in CD4^+^ T cells from the cKO mice compared with that from the WT mice (Figure 4B). The ratio of naïve T cells was decreased and T_CM_ and T_EM_ was increased in CD8^+^ T cells from the cKO mice compared with that from the WT mice (Figure S10C, Supporting Information). The absolute number of CD4^+^ T cells in spleen was decreased in cKO mice compared with WT mice (Figure S10D, Supporting Information), the absolute number of T_CM_ was decreased in CD4^+^ T cells from the cKO mice compared with that from the WT mice (Figure S10E, Supporting Information), and the absolute number of naïve T cells was decreased and T_CM_ and T_EM_ were increased in CD8^+^ T cells from the cKO mice compared with that from the WT mice (Figure S10F, Supporting Information). IFN‐γ secretion by CD4^+^ and CD8^+^ T cells in cKO mice was increased compared with that in WT mice (Figure 4C). The proliferation ability of CD4^+^ T cells was increased in cKO mice (Figure 4D). Although there was no difference between the ratios of Treg subsets and the FOXP3 protein level in cKO mice compared with WT mice (Figure S10G,H, Supporting Information), the inhibitory function of Treg cells was downregulated in cKO mice compared with WT mice (Figure 4E and Figure S10I (Supporting Information)), which is consistent with a previous study showing that SOCS1 is essential for maintaining Treg cell function.^[^ ^25^ ^]^ These data suggested that *Socs1* deficiency in T cells activated T cells and might induce severe GVHD in an HSCT mouse model.

To validate this hypothesis, we examined the effect of *Socs1* deficiency in T cells in a murine GVHD model (C57BL/6 to BALB/c). The results showed that *Socs1* deficiency in T cells exacerbated GVHD symptoms and shortened the life span of GVHD mice (Figure 4F,G and Figure S11A (Supporting Information)). Pathology analysis showed more severe GVHD‐induced target organ tissue damage in the recipient lung, liver, small intestine, and large intestine by T cells from cKO mice than by T cells from WT mice (Figure S11B, Supporting Information). These results demonstrated that *Socs1* deficiency in T cells exacerbated GVHD in mouse models.

### 2.5 *Socs1* Deficiency Disrupted the Protective Role of *G‐CSF* in GVHD Models

**Figure 5:**
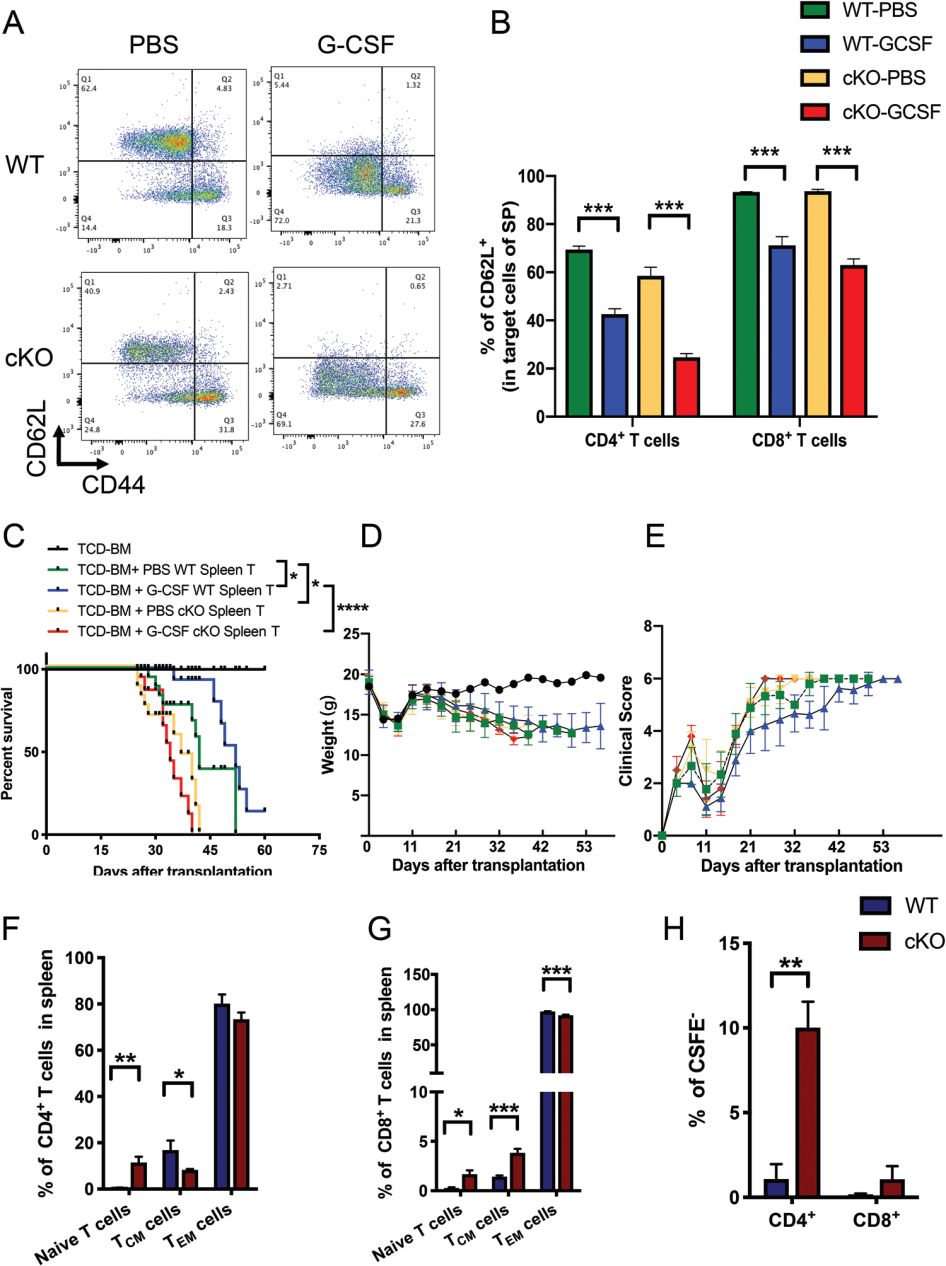
T cell loss of *Socs1* disrupted the protecting role of G‐CSF in GVHD models. A) Representative flow cytometry results show the cell subsets in CD4^+^ and CD8^+^ T cells from the spleens of WT or cKO mice treated with PBS or G‐CSF. B) CD62L expression level on CD4^+^ and CD8^+^ T cells in the spleens of WT or cKO mice treated with PBS or G‐CSF. C) Survival curves of GVHD mouse models. A total of 5 × 10^6^ TCD‐BM from WT mice treated with PBS and 3 × 10^6^ T cells from the spleens of the respective treated donor mice were transplanted into the corresponding wild type recipient mice. 10 mice per group. D) Weight of GVHD mice. E) Clinical score of GVHD mice. F,G) Flow cytometric analysis of the ratio of T cell subsets on donor‐derived CD4^+^ (F) and CD8^+^ (G) T cells in the spleens of recipient mice. H) CFSE analysis showed the proliferation ability of donor‐derived T cells in the spleen of recipient mice. The experiment was repeated at least 3 times, with 4–6 mice per group. Error bars represent the mean ± SEM, ***p < 0.001, **p < 0.01, *p < 0.05.

Our previous results demonstrated that G‐CSF directly upregulated *SOCS1* expression levels in T cells and that SOCS1 played a key inhibitory role in T cell activation. Thus, we hypothesized that SOCS1 is the key mediator in G‐CSF‐induced T cell tolerance. To investigate this hypothesis, we first analyzed the cell subsets in WT and *Socs1* cKO mice after G‐CSF administration. The results showed that G‐CSF inhibited CD62L expression levels in both WT and cKO mice (**Figure** **5**A,B), which is consistent with our previous studies using clinical samples.^[^ ^26^ ^]^ Although ratio of Treg subsets was increased in the WT mice treated with PBS compared with cKO mice treated with PBS (Figure S12A, Supporting Information), the FOXP3 expression level showed no difference between these two groups (Figure S12B, Supporting Information). The increased CD25^+^ T cell populations contributed to the increased ratio of Treg cell subsets in the WT mice treated with PBS compared with cKO mice treated with PBS (Figure S12B,C, Supporting Information), which also indicated that T cells were activated in *Socs1* cKO mice. To further investigate whether *SOCS1* is essential for the protective role of G‐CSF in GVHD, we employed a murine GVHD model (C57BL/6 to BALB/c) in which donor mice were administered PBS or G‐CSF. G‐CSF prolonged the survival of WT mice compared with PBS mice (blue vs green); however, G‐CSF treatment exacerbated GVHD when T cells had *Socs1* deficiency (red vs blue) (Figure 5C–E). *Socs1* deficiency in T cells also exacerbated GVHD (yellow vs green), which is consistent with previous results (Figures 4F and 5C–E). Moreover, we investigated the phenotype of G‐CSF‐administrated donor‐derived T cells in recipient mice. Compared with those in the WT mice, the naïve populations of both CD4^+^ and CD8^+^ T cells in the cKO mice were increased (Figure 5F,G). The proliferation ability of CD4^+^ T cells from cKO mice was significantly increased compared with that of CD4^+^ T cells from WT mice (Figure 5H). It is suggested that high level of naïve compartment and increased proliferation ability of *Socs1* ^−/−^ derived T cells in recipient mice accelerated GVHD damage. Taken together, these results demonstrated that *Socs1* deficiency disrupted the protective role of G‐CSF in murine GVHD models.

### 2.6 SOCS1 Restrained T Cell Activation by Inhibiting CSF3R/JAK2/STAT3 and NLRP3 Inflammasome Signaling

**Figure 6:**
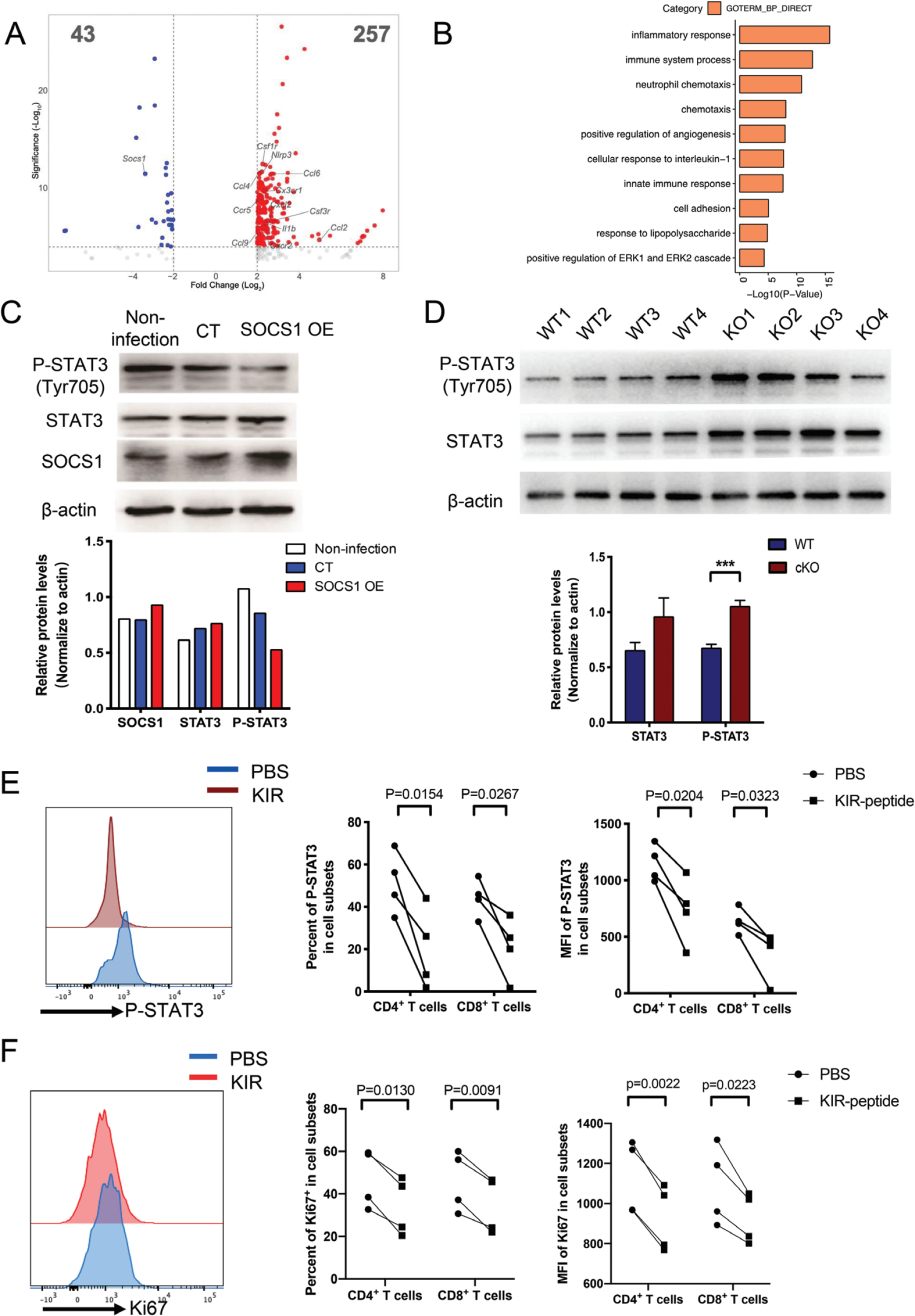
SOCS1 inhibited T cell activation by inhibiting STAT3 phosphorylation. A) RNA‐seq volcano plots comparing gene expression between *Socs1*‐specific knockout T cells and T cells from WT mice (*n* = 3). B) Gene pathway enrichment analysis for the upregulated genes in *Socs1* deficiency T cells. C) Western blot analysis of STAT3 and phosphorylated STAT3 levels in the SOCS1‐overexpressing Jurkat T cell line. D) Western blot analysis of STAT3 and phosphorylated STAT3 levels in the splenic T cell from *Socs1* cKO or WT mice. E) Flow cytometric analysis of the phosphorylated STAT3 levels in SOCS1 mimetic (KIR) or PBS treated primary T cells. *n* = 4. F) Flow cytometric analysis of the Ki67 levels in SOCS1 mimetic (KIR) or PBS‐treated primary T cells. *n* = 4. Error bars represent the mean ± SEM, ***p < 0.001.

To investigate the mechanism by which SOCS1 inhibits T cell activation, we performed RNA‐seq in *Socs1*‐specific knockout T cells and T cells from WT mice. Consistent with previous results that G‐CSF inhibited T cell activation by upregulating *SOCS1* expression level (Figures 1B and 5C), *Socs1*‐specific knockout T cells highly expressed G‐CSF receptor *Csf3r* (**Figure** **6**A), which indicated that SOCS1 inhibited intracellular transduction of G‐CSF signaling pathway by blocking CSF3R. To our surprise, we also observed many other inflammatory signaling related genes such as *Csf1r, Il‐1b*, and *Nlrp3* were activated in *Socs1*‐specific knockout T cells compared with T cells from WT mice (Figure 6A), which indicated the NLRP3 inflammasome signaling was activated in *Socs1*‐specific knockout T cells. GO analysis also showed that the inflammatory response and IL‐1 signaling pathway related genes were enriched in *Socs1* deficiency T cells compared with WT T cells (Figure 6B). These results indicated that SOCS1 not only acting as an important negative regulator for G‐CSF signaling pathway, but also effectively inhibited NLRP3 inflammasome singling pathways in T cells.

Furthermore, we detected the regulation role of SOCS1 in JAK/STAT signaling pathway by western blot. Western blotting analysis of a SOCS1‐overexpressing Jurkat T cell line showed that a high SOCS1 expression level inhibited the phosphorylation of STAT3 (Figure 6C). In addition, we detected STAT3 protein levels in splenic T cells and found that the phosphorylation of STAT3 was upregulated in T cells from cKO mice compared with those from WT mice (Figure 6D). Previous studies developed a peptide corresponding to the KIR domain of SOCS1 that directly binds to the phosphorylation site of JAK2 and inhibits JAK2 kinase activity.^[^ ^27^ ^]^ Therefore, we employed this mimetic to treat human primary T cells in vitro. P‐STAT3 was also inhibited in KIR‐peptide‐treated CD4^+^ T cells and CD8^+^ T cells compared with T cells from the PBS treatment group (Figure 6E). Moreover, we found that KIR peptide treatment decreased Ki67 levels in both CD4^+^ T cells and CD8^+^ T cells compared with T cells from the PBS treatment group (Figure 6F). These results indicated that mimetic of SOCS1 functional peptide could restrain T cell activation by inhibiting JAK2/STAT3 signaling pathway.

### 2.7 The *SOCS1* Expression Level in Primary T Cells Was Negatively Related to GVHD Occurrence after HSCT

**Figure 7:**
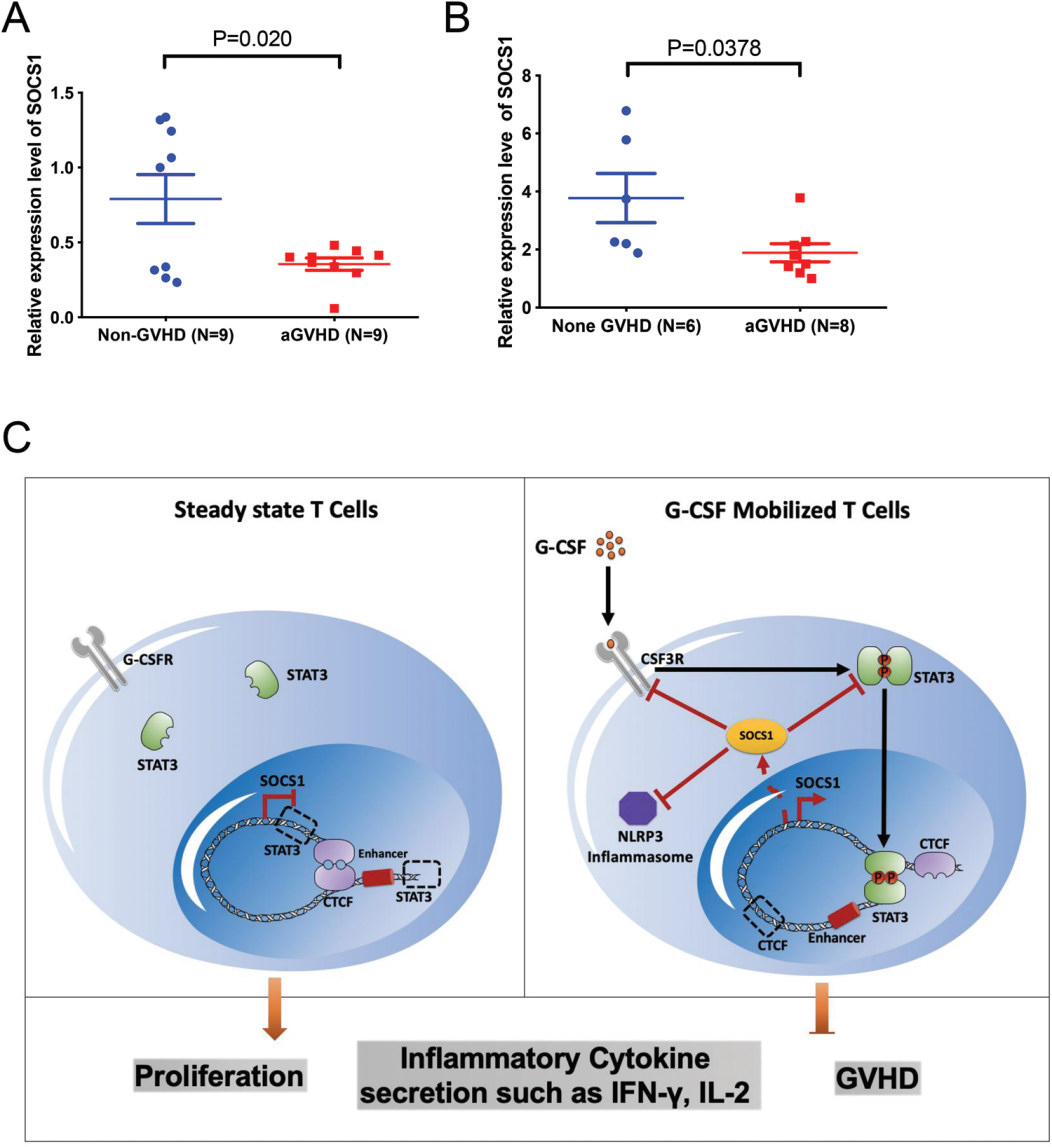
*SOCS1* expression level in primary T cells is negatively related with GVHD occurrence after HSCT. A) Quantitative real‐time PCR showed the expression level of *SOCS1* in CD4^+^ T cells from peripheral allografts and the aGVHD occurrence in related patients. B) *SOCS1* expression level in CD3^+^ T cells from patients with aGVHD and patients without aGVHD in the same period after allo‐HSCT. C) Schematic summary of SOCS1 restraining T cell activation in G‐CSF mobilized T cells. G‐CSF activated STAT3 by receptor CSF3R, then phosphorylated STAT3 entered into nucleus and upregulated *SOCS1* expression level by reorganizing chromatin structure around *SOCS1* locus. High expression level of *SOCS1* inhibited T cell proliferation, inflammatory cytokine secretion and abolished GVHD by inhibiting CSF3R, STAT3, and NLRP3 inflammasome activation.

The previous results showed that SOCS1 tightly controlled T cell tolerance and inhibited GVHD. To further investigate the clinical correlation between SOCS1 and GVHD, we detected the expression level of *SOCS1* in CD4^+^ T cells from peripheral blood allografts and monitored for the occurrence of GVHD after HSCT for 2 years. The results showed that low expression levels of *SOCS1* in *CD4^+^* T cells from peripheral blood allografts correlated with aGVHD occurrence in patients after allo‐HSCT but not in those without aGVHD (**Figure** **7**A). We also assessed the relationship between the expression level of *SOCS1* in T cells and aGVHD occurrence in patients after allo‐HSCT. The results showed that there was a lower expression level of *SOCS1* in the patients with aGVHD than in the patients without aGVHD at the same timepoint after allo‐HSCT (Figure 7B). These results indicated that a low expression level of *SOCS1* in T cells might corelate with aGVHD occurrence after allo‐HSCT.

## 3 Discussion

In this study, we systematically investigated the dynamics of transcriptomes and 3D chromatin structures in T cells in states ranging from a steady state to a G‐CSF‐induced hyporesponsive state and found that G‐CSF directly upregulated *SOCS1* expression levels by reorganizing the chromatin structure at the *SOCS1* locus. In vitro experiments demonstrated that elevating *SOCS1* in human primary T cells with lentivirus inhibited T cell activation, which inhibited T cell proliferation and elevated the expression level of the T cell exhaustion marker TIGIT. Previous study showed that higher expression level of TIGIT in patients after allo‐HSCT was associated with a decreased incidence of acute GVHD.^[^ ^28^ ^]^ In GVHD mouse models, TIGIT‐Fc‐treated mice had alleviated GVHD symptom occurrence and delayed mortality compared to that in isotype control group mice.^[^ ^29^ ^]^ Taken together, SOCS1‐mediated increased expression of TIGIT in primary T cells suggests that high expression level of SOCS1 decreased GVHD occurrence might through upregulating TIGIT in patients after allo‐HSCT. In in vivo experiments, mouse models with T cell‐specific knockout *Socs1* demonstrated that loss of *Socs1* in T cells enhanced T cell proliferation ability, increased secretion of the proinflammatory cytokine IFN‐γ, exacerbated GVHD‐induced target organ damage, and shortened the life span of GVHD mice. Moreover, a lack of *Socs1* in T cells disrupted the protective role of G‐CSF in GVHD mouse models and accelerated the death of GVHD mice. Mechanismly, SOCS1 inhibited G‐CSF/JAK/STAT signaling pathway not only by inhibiting phosphorylation of STAT3, but also blocking CSF3R in a negative feedback loop. Furthermore, SOCS1 also inhibits NLRP3 inflammasome signaling to restrain T cell activation (Figure 7C). These data indicated the essential role of SOCS1 in abolishing inflammatory response involved T cell activation and SOCS1 could be a potential target for GVHD prevention and treatment.

G‐CSF, as a mobilizer of hematopoietic stem/progenitor cells, has been widely used in healthy donors before HSCT. Substantial studies have revealed the immunoregulatory effects of G‐CSF on both innate immune cells and adaptive immune cells, especially T cells.^[^ ^19^, ^30^ ^]^ Previous mechanistic studies in myeloid cells revealed that G‐CSF activated JAK/STAT signaling and further induced *SOCS3* expression, which acted as an important negative regulator of the G‐CSF response by preventing JAK activation.^[^ ^31^ ^]^ The mechanism by which G‐CSF regulates T cell tolerance has been speculated to be similar to that in myeloid cells.^[^ ^18b^ ^]^ In our study, we found that *SOCS1*, not *SOCS3*, was upregulated in G‐CSF‐administered CD4^+^ T cells and CD8^+^ T cells by RNA‐seq. Integration of transcriptome and epigenome analysis results showed that STAT3 acted as a structural protein that mediated reorganization of the chromatin structure around the *SOCS1* locus by mediating new loop formation between the *SOCS1* promoter and upstream enhancer and upregulated the *SOCS1* expression level. Further investigation showed that SOCS1 not only inhibited the phosphorylation of STAT3 protein, but also inhibited CSF3R expression level, which indicated that SOCS1 simultaneously inhibits STAT3 activation and blocks G‐CSF receptor in a negative feedback loop to avoid comprehensive G‐CSF/JAK/STAT signaling activation in T cells after G‐CSF administration. In addition, we also found that PRDM1, an important repressive TF that has been proven to be essential for maintaining T cell homeostasis,^[^ ^32^ ^]^ was upregulated and mediated loop gains in T cells after G‐CSF administration. This finding might indicate that PRDM1 also contributes to G‐CSF‐induced T cell hyporesponsiveness. Taken together, our systematic multiomics analysis provides new insight into the transcriptome and epigenome landscape in G‐CSF‐induced hyporesponsive T cells.

Accumulated evidence has demonstrated the important role of SOCS1 in autoimmune disease. The role of SOCS1 in regulating T cell activation depends on the context. For example, *SOCS1* expression level could be elevated by IL‐6/STAT3 signaling pathway, whereas high level of SOCS1 does not negatively feedback inhibit IL‐6/STAT3 signaling, but rather inhibits IFN‐γ/STAT1 signaling pathway and suppresses Th1 differentiation, that is, SOCS1 induces Th17 generation in IL‐6/STAT3‐mediated Th17 differentiation process.^[^ ^33^ ^]^ Therefore, T cell specific knockout *Socs1* could protect mice from EAE (a Th17‐dependent autoimmune disease model).^[^ ^33b^ ^]^ Recently, using whole exome/genome sequencing, Hadjadj et al. identified germline loss‐of‐function mutations in the *SOCS1* gene related to early onset autoimmune manifestations.^[^ ^34^ ^]^ SOCS1 was also identified as a checkpoint for antigen‐experienced CD4^+^ T cell expansion.^[^ ^35^ ^]^ However, the role of SOCS1 in another immune‐related disorder, GVHD, has not been elucidated. Our clinical data showed that a low expression level of *SOCS1* in T cells from patients was correlated with GVHD occurrence after HSCT. Murine models demonstrated that *Socs1* deficiency in T cells activated T cell proliferation and IFN‐γ secretion, leading to exacerbation of GVHD and a shortened life span of recipient mice. Our data also showed that SOCS1 inhibited several inflammatory signaling pathway including JAK2/STAT3 signaling and NLRP3 inflammasome signaling. NLRP3 inflammasome signaling is critical for innate immune components that orchestrate host immune homeostasis, following studies showing that this protein complex was also found to be present in T and B lymphocytes.^[^ ^36^ ^]^ NLRP3 inflammasome signaling can be primed by signal 1 which is Toll‐like receptors leading to NF‐κB mediating gene transcription and synthesizing the IL‐1β precursor pro‐IL‐1β and NLRP3.^[^ ^36a^ ^]^ Then, NLRP3 inflammasome signaling fully activated by signal 2 and produced bioactive IL‐1β.^[^ ^36a^ ^]^ Here, we found both *Il‐1b* and *Nlrp3* were upregulated in *Socs1*‐specific knockout T cells, which indicated that NLRP3 inflammasome signaling fully activated in *Socs1* deficiency T cells. These data demonstrated SOCS1 inhibited T cell activation by blocking several inflammatory signaling pathways. Therefore, directly elevating SOCS1 expression is a potential strategy for the prevention and treatment of GVHD.

SOCS3 is another important member of the SOCS family, and *Socs3* deficiency has been proved to promote aGVHD‐related mortality in mouse models.^[^ ^37^ ^]^ Previous structural analysis showed that SOCS1 could directly inhibit the catalytic activity of JAK1, JAK2, and tyrosine kinase 2 (TYK2), indicating that inhibition of JAK/STAT signaling by SOCS1 is an order of magnitude more potent than that by *SOCS3*.^[^ ^14b^ ^]^ Therefore, as we indicated a key regulatory role of SOCS1 in GVHD, SOCS1 should be considered a promising target for clinical treatment of GVHD. In our study, we treated human primary T cells with a SOCS1 mimetic, a peptide corresponding to the KIR domain of SOCS1 that binds to the phosphorylation site of JAK2 and inhibits JAK2 kinase activity.^[^ ^27^ ^]^ The results showed that the SOCS1 mimetic could inhibit STAT3 phosphorylation and T cell proliferation, indicating that the KIR peptide is an alternative tool for increasing SOCS1 levels. However, a high dose (500 × 10^−6^ m) of KIR peptide is required to effectively inhibit STAT3 phosphorylation in vitro. Thus, optimization of the KIR peptide to reduce the effective dose is needed for further application in GVHD treatment in vivo.

Collectively, by multiomics analysis of the transcriptomes and chromatin structures of G‐CSF‐induced hyporesponsive T cells, we found that STAT3 reorganized the chromatin structure around the *SOCS1* locus and upregulated the *SOCS1* expression level. Elevation of SOCS1 inhibited T cell activation, and loss of SOCS1 activated T cells and promoted GVHD‐induced mortality. Together with the negative relationship between the *SOCS1* expression level in T cells from patients and GVHD occurrence, these results suggest that upregulating SOCS1 levels might represent a future target for prophylaxis and treatment of GVHD.

## 4 Experimental Section

### Samples

Bone marrow was collected from healthy donors. Peripheral blood was collected from 14 patients with or without aGVHD after allo‐HSCT (Table S1, Supporting Information). Peripheral grafts and bone marrow grafts were collected from 18 healthy donors between 1 November 2018 and 31 December 2018, and the related allo‐HSCT patients were followed up to 30 October 2020 (Table S2, Supporting Information). Human bone marrow mononuclear cells (BMMCs) or peripheral blood mononuclear cells were isolated by Ficoll density centrifugation. The study was approved by the Ethics Committee of Peking University People's Hospital (2017PHB033‐01), and written informed consent from all subjects was obtained in accordance with the Declaration of Helsinki.

### Mice

*LckCre*‐*Socs1^fl/fl^* (T cell‐specific *Socs1*‐cKO) and *Socs1^fl/fl^* (littermate control; WT) mice (sex‐ and age‐matched) were used. All mice were maintained in the specific pathogen‐free animal facility of Peking University People's Hospital. All experiments were performed with approval according to the National Institutes of Health's Guide for the Care and Use of Laboratory Animals (2020PHB067).

### T Cell Isolation and Culture

Human BMMCs were isolated from the BM of healthy donors before and after in vivo G‐CSF application by Ficoll density centrifugation. CD3^+^ T cells were purified by positive selection (CD3 MACS MultiSort beads; Miltenyi Biotec, Bergische Gladbach, Germany). The isolated CD3^+^ T cells were cultured in IMDM (Iscove's Modified Dulbecco's Medium, Gibco, Invitrogen, Carlsbad, CA) containing 10% BIT 9500 (Stemcell Technologies, Vancouver, CA) and stimulated with Dynabeads Human T‐Activator CD3/CD28 (Gibco, Invitrogen, Carlsbad, CA).

### Lentivirus‐Mediated *SOCS1* Overexpression in T Cells

The *SOCS1*‐overexpressing lentivirus was purchased from Sangon Biotech (Shanghai, China). CD3^+^ T cells were prestimulated for 24 h with Dynabeads Human T‐Activator CD3/CD28 in IMDM containing 10% BIT 9500, and recombinant human Interleukin‐2 (rhIL‐2) was added at a dose of 100 U mL^−1^. After 24 h, the cells were transduced with thawed lentiviruses that were added directly to the plate. Then, 6 µg mL^−1^ polybrene (Sigma, USA) was added. The cells were incubated for another 24 h at 37 °C and 5% CO_2_, and fresh medium was changed. Green fluorescent protein positive (GFP^+^) cells were isolated after a 72 h infection and cultured in IMDM containing 10% BIT 9500 with rhIL‐2 routinely used.

### Flow Cytometric Analysis

Surface staining was performed with directly conjugated monoclonal antibodies for 20 min at room temperature for human samples. The cells were washed and resuspended in PBS before flow cytometric analysis. The monoclonal antibodies used were anti‐human CD4–Percp‐Cy5.5/APC‐H7, CD8–APC (allophycocyanin)‐R700/V500, PD‐1–PE (phycoerythrin)–Cy7, TIM*‐3*–APC, TIGIT–BV605, 2B4–AF700, and CD160–PE (BD Bioscience San Diego, CA, USA). Intracellular staining was carried out by using a fixation/permeabilization kit (BD Bioscience) after resuspension according to the manufacturer's instructions. Ki67–PE (BD Pharmingen) was added and incubated for 20 min at room temperature.

The surfaces of mouse samples were stained with direct‐conjugated monoclonal antibodies for 30 min at 4 °C. After incubation, the cells were washed and resuspended in phosphate‐buffered saline before flow cytometry analysis. The monoclonal antibodies used were anti‐mouse CD3–Percp, CD4–APC‐H7, CD8–FITC (fluorescein), CD44–PE–Cy7, and CD62L–APC (BD Bioscience San Diego, CA, USA). Detailed antibody information was listed in Table S3 (Supporting Information).

### Cytokine Detection by Flow Cytometry

T cells were stimulated with Dynabeads Human/Mouse T‐Activator CD3/CD28 (Gibco, Invitrogen, Carlsbad, CA). After 72 h of culture, GolgiPlug (BD Pharmingen, San Diego, CA, USA) was added for 4 h. Cells were harvested for surface staining as described above. Intracellular staining was carried out by using a fixation/permeabilization kit (BD Bioscience) after resuspension according to the manufacturer's instructions. For human samples, IL‐2–V450, IFN‐γ–BV510, IL‐17–PE, IL‐4–APC, and IL‐10–PE (BD Pharmingen) were added and incubated for 20 min at room temperature. For mouse samples, IFN‐γ–PE (BD Pharmingen) was added and incubated for 30 min at 4 °C. Detailed antibody information was listed in Table S3 (Supporting Information).

### RT‐PCR

RNA was extracted using the RNeasy Mini Kit (Qiagen, 74106) according to the manufacturer's protocol. For quantitative PCR, first strand synthesis was performed using a complementary DNA (cDNA) reverse transcription kit (TaKaRa, RR047A) according to the manufacturer's protocol. Quantitative PCR assays were performed in 96‐well MicroAmp Fast Optical 96‐Well Reaction Plates (Applied Biosystems, 4344904) using SYBR Green (Roche, 04913914001). Signals were detected using a 7500 Real‐Time PCR System (Applied Biosystems). Target gene cycle numbers were normalized to the housekeeping gene *18S* to obtain the ΔCT values. The 2^–ΔΔCT^ method was used. The primer sequences were as follows: *SOCS1* forward: 5ʺ‐CACGCACTTCCGCACATTC‐3ʺ; *SOCS1* reverse: 5ʺ‐TAAGGGCGAAAAAGCAGTTCC‐3ʺ; human *18S* forward: 5ʺ‐ACCGATTGGATGGTTTAGTGAG‐3ʺ; and human *18S* reverse: 5ʺ‐CCTACGGAAACCTTGTTACGAC‐3ʺ.

### GVHD Mouse Models

Acute GVHD was induced as described previously.^[^ ^20b^ ^]^ In brief, WT or *Socs1* cKO donor mice were sacrificed the day after the last dose was given. Splenic T cells were isolated by negative selection using a Pan T Cell Isolation Kit II (Miltenyi‐Biotec, Germany), and the obtained cells had a purity of >95%. BM cells from WT mice were T cell‐depleted with anti‐CD90.2 MicroBeads (Miltenyi‐Biotec). BALB/c recipient mice received 8 Gy total body irradiation, and 3 × 10^6^ or 2 × 10^6^ T cells from the spleen of cKO mice or WT mice were transplanted intravenously the following day. 5 × 10^6^ T cell‐depleted bone marrow cells (TCD‐BM) were transplanted as protective cells from the WT group donor mice to all groups of recipient mice.

### G‐CSF‐Administrated GVHD Mouse Models

Donor cKO or WT mice (C57BL/6 background) were subcutaneously injected with *G‐CSF* (250 µg kg^−1^ daily) or the same volume of PBS for 5 days. These donor mice were sacrificed the day after the last dose was given. Splenic T cells were isolated from G‐CSF‐ or PBS‐treated donor mice by negative selection using a Pan T Cell Isolation Kit II (Miltenyi‐Biotec, Germany), and the obtained cells had a purity of >95%. BM cells from PBS‐treated WT mice were T cell‐depleted with anti‐CD90.2 MicroBeads (Miltenyi‐Biotec). Once splenic T cells and TCD‐BM cells were purified, they were injected into the tail veins of prepared recipient mice. BALB/c hosts were subjected to total body irradiation from a [^60^Co] source (8 Gy). They were randomly grouped.

### T‐Cell Proliferation Analysis (CFSE)

CD3^+^ spleen T cells were isolated with a pan T cell isolation kit (Miltenyi‐Biotec, Germany). The purified T cells were stained with CFSE (carboxyfluorescein succinimidyl ester, BD Bioscience) to achieve a final concentration of 5 × 10^−6^ m for 5 min at 37 °C and washed twice with complete medium with 10% FBS (fetal bovine serum). Labeled T cells (2 × 10^5^ cells per well) were stimulated with CD3/28 beads in flat‐bottomed 96‐well plates in complete RPMI (roswell park memorial institute) 1640. After 96 h, cells were harvested, stained with CD4–APC‐H7 and CD8–APC‐R700 (BD Bioscience), and then analyzed by flow cytometry. Detailed antibody information was listed in Table S3 (Supporting Information).

### Treg Suppression Assay

CD3^+^ spleen T cells were isolated with Pan T cell isolation kit (Miltenyi, 130‐095‐130). CD4^+^CD25^−^ Teff and CD4^+^CD25^+^ Tregs were sorted by flow cytometry. Freshly sorted Teffs were stained with 5 × 10^−6^ m CFSE (BD Bioscience) for 5 min at 37 °C. 5 × 10^4^ Teff cells were cocultured with Tregs at Teff/Treg ratios of 1:1, 2:1, and 4:1 in the presence of Dynabeads Mouse T‐Activator CD3/CD28 beads (Invitrogen, 11452D) and 30 U mL^−1^ rhIL‐2 in RPMI complete medium (Gibco, Invitrogen). CFSE dilution was analyzed by flow cytometry after 72 h of culture. Detailed antibody information was listed in Table S3 (Supporting Information).

### SOCS1 Mimetic Treatment

*SOCS1 KIR* domain mimetic peptide (sequence: DTHFRTFRSHADYRRI) was synthesized in GUOPING PHARMACEUTICAL Company (Anhui, China). Peptide was dissolved in PBS. Human primary CD3^+^ T cells were prestimulated with Dynabeads Human T‐Activator CD3/CD28 beads (Invitrogen, 11161D) and 200 U mL^−1^–rhIL‐2 in IMDM complete medium (Gibco, Invitrogen) for 72 h in 6‐well plates (1 × 10^6^ mL^−1^). To activate the JAK/STAT signaling pathway, T cells were then incubated with 100 ng mL^−1^ IFN‐α (Cell Signaling Technology, 8927SC). Meanwhile, SOCS1 peptide was added at a final concentration of 500 × 10^−6^ m and cultured for 90 min. STAT3 phosphorylation and Ki67 expression level were detected by flow cytometry. Detailed antibody information was listed in Table S3 (Supporting Information).

### RNA‐Seq Experiments and Analysis

Total mRNA with a polyA tail was extracted and reverse transcribed to cDNA for sequencing. Three biological repeats were performed for each sample, and 20 million reads were sequenced for each repeat. The sequenced reads were mapped to the human reference genome (hg19) by TopHat2,^[^ ^38^ ^]^ and gene expression was quantified by Cufflinks.^[^ ^39^ ^]^ RStudio software was used for the downstream statistical analyses.

### ATAC‐Seq Experiments and Analysis

The ATAC‐seq experiment was performed following Buenrostro et al.’s protocol.^[^ ^40^ ^]^ Two biological repeats were used for each sample, and 20 million reads were sequenced for each repeat. The sequenced reads were mapped to the human reference genome (hg19) by Bowtie2,^[^ ^38^ ^]^ and peak signals were quantified by MACS2 and deepTools. RStudio software was used for the downstream statistical analyses.

### Hi‐C Experiments

The cells were resuspended in fresh PBS. Cell counts were performed. Then, a cell suspension with a final concentration of 1 × 10^6^ cells per 1 mL of PBS was prepared. A total of 1 × 10^6^ cells was isolated and cross‐linked with 1% formaldehyde for 10 min at room temperature, and then, 2.5 m glycine solution was added to a final concentration of 0.2 m. Then, the cells were collected, flash‐frozen in liquid nitrogen, and stored at −80 °C. The Hi‐C experiment was performed following the in situ Hi‐C protocol.^[^ ^21c^ ^]^

### Hi‐C Data Analysis

Read mapping and filtering of the Hi‐C data were performed following previous methods.^[^ ^41^ ^]^ All Hi‐C sequencing reads were mapped to the human reference genome (hg19) using Bowtie2.^[^ ^42^ ^]^ The two ends of the paired‐end reads were mapped independently using the first 36 bases of each read. Redundant and nonuniquely mapped reads were filtered out and the reads within 500 bp upstream of enzyme cutting sites (Mbol) were kept for size selection. The iterative correction and eigenvector decomposition method^[^ ^43^ ^]^ was used to normalize raw interaction matrices.

### Statistical Analyses

All the results were shown as mean ± standard error of the mean (SEM). Student's *t* test was used for two groups’ analyses. One‐way analysis of variance was used to compare the means of more than two groups. *p values <0.05 were considered to be significant. Statistical analyses were performed on GraphPad 8.0 software.

## Conflict of Interest

The authors declare no conflict of interest.

## Author Contributions

H.G. and R.L. contributed equally to this work. Y.‐J.C., C.L., and X.‐J.H. designed the study; H.G. and R.L. collected data; H.G. and M.W. performed the in vivo mouse model experiments and ex vivo experiments; R.L. analyzed the RNA‐seq, ATAC‐seq, and Hi‐C data; H.G., R.L., Y.‐J.C., C.L., and X.‐J.H. drafted the paper; all authors contributed to data interpretation and paper preparation and approved the final version.

## Supporting information

Supporting Information

Supporting Information

## Data Availability

The data that support the findings of this study are openly available in Genome Sequence Archive at https://ngdc.cncb.ac.cn/gsa‐human/, reference number HRA000625. The Gene Expression Omnibus (GEO, https://www.ncbi.nlm.nih.gov/gds/) accession number for the Hi‐C data of GM12878 in the public domain is GSE63525. The Encyclopedia of DNA Elements (ENCODE, https://www.encodeproject.org) accession number for the ChIP‐seq data of GM12878 in the public domain are ENCSR000DZN (CTCF), ENCSR000DZV (STAT3), ENCSR000EUM (YY1), ENCSR000ARD (EZH2), ENCSR000AKC(H3K27ac) and ENCSR000DRY (H3K4me3).
