# Supplementary material for: Multiomics Analysis Identifies SOCS1 as Restraining T Cell Activation and Preventing Graft‐Versus‐Host Disease
Source: Adv Sci (Weinh). 2022 May 18;9(21):2200978. doi: 10.1002/advs.202200978 (PMC9313503; doi:10.1002/advs.202200978)
Supplement: Supplementary file 1 — Supporting Information [file ADVS-9-2200978-s002.pdf]

ADVANCED SCIENCE
Open Access

Supporting Information

for *Adv. Sci.*, DOI 10.1002/advs.202200978

Multiomics Analysis Identifies SOCS1 as Restraining T Cell Activation and Preventing Graft-Versus-Host Disease

Huidong Guo, Ruifeng Li, Ming Wang, Yingping Hou, Shuoshuo Liu, Ting Peng, Xiang-Yu Zhao, Liming Lu, Yali Han, Yiming Shao, Ying-Jun Chang, Cheng Li* and Xiao-Jun Huang**

ADVANCED SCIENCE
Open Access

Supporting Information

for *Adv. Sci.*, DOI: 10.1002/advs.202200978

Multiomics Analysis Identifies SOCS1 as Restraining T Cell Activation and Preventing Graft-Versus-Host Disease

Huidong Guo, Ruifeng Li, Ming Wang, Yingping Hou, Shuoshuo Liu, Ting Peng, Xiang-Yu Zhao, Liming Lu, Yali Han, Yiming Shao, Ying-Jun Chang, Cheng Li,* and Xiao-Jun Huang**

Supporting Information

Supplementary materials and methods

A/B compartment analysis

We used ICE-normalized interaction matrices at 500 kb resolution to detect chromatin compartment types with the R package HiTC.[1] Positive or negative values of the first principal component separated the chromatin regions into two spatially segregated compartments. The compartment with a higher gene density was assigned as the A compartment, and the other compartment was assigned as the B compartment.[2]

TAD analysis

We used ICE-normalized interaction matrices at 40 kb resolution to call TAD by a Perl script, matrix2insulation.pl (https://github.com/blajoie/crane-nature-2015). A higher resolution was possible because TADs are smaller than A/B compartments. Insulation scores (IS) were calculated for each chromosome bin, and the valleys of the IS identified the TAD boundaries. TADs smaller than 200 kb or located in telomeres/centromeres were filtered out as in previous methods.[3] In comparisons of TADs between two cell lines, at least 70% overlap between two TADs was considered to indicate TAD conservation.[4] We used BEDtools with the option of "intersectBed −f 0.70 – r" to identify conserved TADs.[5]

Gene ontology analysis

We used DAVID Bioinformatics Resources 6.7 for Gene Ontology analysis.[6] The set of all human genes was used as the background gene list.

Chromatin immunoprecipitation

Jurkat cells were fixed in 1% formaldehyde (Sigma-Aldrich, F8775) for 10 min at 37 °C. Subsequently, glycine was added to 125 mM and incubated at 37 °C for 5 min at 37 °C. Next, the cells were pelleted and washed twice with cold PBS. The pellets were stored at −80 °C until use.

Nuclei from 10 M cells per ChIP-seq were extracted, and chromatin was sonicated with a Bioruptor Sonication Device. Immunoprecipitation reactions were performed overnight with STAT3 (Cell Signaling Technology, 9139S, MA), H3K27ac or CTCF (ABclonal, A1133, China) antibodies. The next morning, antibodies and chromatin

were captured using Protein G Dynabeads (Thermo Fisher). The material was washed, eluted and treated with RNase A for 30 min at 37 °C and proteinase K for 3 h at 65 °C.

Library preparation and sequencing

Library preparation from ChIP-seq DNA was performed using the Ultra II Library Prep Kit (NEB E7103L) and Multiplex Oligos for Illumina (NEB E7335L) and sequenced on an Illumina NextSeq 2500 instrument (150 base pairs single end).

ChIP-seq data processing, heat map generation, and edgeR analysis

H3K27ac, CTCF, and STAT3 ChIP-seq analyses were performed with an average range of 20–25 × 10^6 reads per independent ChIP-seq experiment. ChIP-seq reads were mapped to the hg19 genome with Bowtie2 using default parameters. Aligned reads were filtered for a minimum MAPQ of 30, and duplicates were removed using SAMtools. Signal tracks were generated by first using BEDTools to produce bedGraph files scaled to 10 million reads per data set. Then, the UCSC Genome Browser utility bedGraphToBigWig was used with default parameters to generate bigwig files. Peaks were called using MACS2 with default parameters. Heat maps of the ChIP-seq signal profiles were generated with the HOMER (http://biowhat.ucsd.edu/homer/index.html) tool annotatePeaks with the following parameters: -ghist 50, -size 10000. ChIP-seq peaks exhibiting differential H3K27ac or STAT3 signals across the time course were identified using edgeR, similar to the process described above.

CUT&Tag experiments and analysis

The CUT&Tag experiments were performed as previously described[7] (Vazyme TD901 kit) to generate DNA libraries derived from human CD8 TpreG cells. We used the SEACR peak caller (http://seacr.fredhutch.org), which was expressly designed for CUT&RUN and CUT&Tag data, to call peaks.

***In vitro* stimulation with G-CSF**

$CD3^+$ T cells isolated from healthy donors and cultured overnight with in IMDM containing 10% BIT 9500, and rhIL-2 was added at a dose of 100 U/ml. Then T cells were incubated with G-CSF (100 ng/ml) for 4 h to detect CSF3R, CD25, CD69, P-STAT1, P-STAT3 and P-STAT5 level, or incubated with G-CSF (100 ng/ml) for 72

h to detect IL-2 secretion level at 37°C and 5% CO_2.

Supplementary Figure Legends

Figure S1. RNA-seq analysis of $CD4^+$ or $CD8^+$ T cells before and after G-CSF administration.

(A) The top ten genes with most upregulated and most downregulated expression in CD8 T_{postG} compared with CD8 T_{preG} were identified (red: higher expression; blue: lower expression). **(B)** Volcano plot comparing CD4 T_{preG} and CD4 T_{postG}. The X-axis shows the fold change (log2). Among the genes, 52 genes were significantly upregulated, and 85 genes were significantly downregulated. **(C)** Corresponding to FigureS1B, the top ten genes with high and low expression were identified (red: high expression; blue: low expression). **(D)** Gene pathway enrichment analysis for the upregulated (red) and downregulated (blue) genes in CD4 T_{postG} cells.

Figure S2. ATAC-seq analysis of $CD4^+$ or $CD8^+$ T cells before and after G-CSF administration.

(A) Volcano plot comparing chromatin accessibility changes in CD8 T_{preG} and CD8 T_{postG}. **(B)** Motif results predicted by HOMER software to have increased chromatin accessibility in CD8 T_{postG} compared with CD8 T_{preG} cells. **(C)** Motif results predicted to have decreased chromatin accessibility in CD8 T_{postG} compared with CD8 T_{preG} cells. **(D)** Motif results predicted upregulated chromatin accessibility in CD4 T_{postG} compared with CD4 T_{preG} cells using ATAC-seq data by HOMER software. **(E)** Motif results predicted downregulated chromatin accessibility in CD4 T_{postG} compared with CD4 T_{preG} cells using ATAC-seq data by HOMER software.

Figure S3. Hi-C analysis of $CD4^+$ or $CD8^+$ T cells before and after G-CSF administration.

(A) High-resolution maps of 3D genome structures. Top left: Whole-genome Hi-C interaction matrix of CD8 T_{preG} cells. Bottom right: Hi-C interaction matrix of chromosome 16 of CD8 T_{preG} cells. Right: Examples of three loop structures. **(B)** The

number of loops. **(C)** The length of the loops. Consistent with previous reports, the median loop length was 185 kb (red dotted line), and the loop length of the CD4 cells increased significantly (median length: CD8 T_{preG} cells, 190 kb; CD8 T_{postG} cells, 180 kb; CD4 T_{preG} cells, 210 kb; CD4 T_{postG} cells, 340 kb). **(D)** A/B compartments of different cell types. The A/B compartments of chromosome 10 inferred from Hi-C data of various samples. **(E)** Genome-wide proportions of A/B compartment changes among two cells before and after G-CSF mobilization (Fisher's exact test < 2.2e-16). **(F)** Boxplots of expression changes of genes grouped by their A/B compartment changes (t-test). **(G)** Read count per million mapped reads around the TAD boundaries using CTCF, H3K27ac ChIP-seq and ATAC-seq data. The X axis represents the distance (kb) from the TAD boundary (B). **(H)** The length of the TADs in CD4 and CD8 cells (t-test).

Figure S4. TADs and Loops Structures are Influenced by G-CSF.

(A) Highly expressed genes in the loop anchor region of CD8 T_{postG} compared to CD8 T_{preG}. Of the 55 upregulated genes, 29 were located in the anchor region of the loop. From the outer circle to the inner circle: ①: gene name; ②: gene expression levels in CD8 T_{postG} (from RNA-seq data); ③: chromosome accessibility from ATAC-Seq in CD8 T_{postG}; ④ red lines: chromatin loops overlap with genes. **(B)** Venn diagram of CD8 T_{preG} and CD8 T_{postG} chromatin loops. APA analysis was performed on the three types of loops in the Venn diagram to verify the reliability of each type of loop. **(C)** Venn diagram of CD4 T_{preG} cells and CD4 T_{postG} cells in chromatin loops. APA analysis was performed on the three types of loops in the Venn diagram to verify the reliability of each type of loop. **(D)** Effects of different chromatin states on gene expression. The boxplot on the left shows the effect of in situ chromatin status on genes. The boxplot on the right represents the effect of chromatin interactions on long-distance gene expression. Enhancers activate gene expression more than the other two chromatin states.

Figure S5. STAT3 and CTCT are colocalized in the whole genome, and STAT3 mediates the spatial interaction between enhancers and promoters.

(A-B) UCSC browser views showing histone modifications and transcription factor (TF) binding sites of *SOCS1* **(A)** and *TXNIP* **(B)** in Jurkat and CD8 T_{preG} cells determined using ChIP-seq and CUT&Tag data. The pink dotted boxes represent sites where the two transcription factors are colocalized. **(C)** Heatmaps displaying STAT3 and CTCF colocalization across the whole genome (the top 5000 CTCF peaks) in GM12878. **(D-E)** Heatmaps displaying STAT3 occupancy at enhancers **(D)** and active promoters **(E)** (top 5000 STAT3 peaks) in GM12878. **(F)** The peaks of STAT3 binding are classified into three clusters. The first cluster includes both enhancer and promoter signals. The second kind of enhancer signal is stronger. The third kind of promoter signal is relatively strong. **(G)** Signal strength of the three kinds of peaks. **(H)** Heatmap of the interaction between Cluster 2 and Cluster 3 in space. The spatial interaction between enhancers and promoters. **(I)** A random selection of the same number of enhancers and promoter peaks has no spatial interaction. **(J)** Immunofluorescence staining of CTCF and STAT3 in Jurkat cells.

Figure S6. G-CSF directly upregulates *SOCS1* expression level in human primary T cells *in vitro*.

(A) The *SOCS1* expression level was determined by quantitative real-time RT-PCR upon G-CSF treatment *in vitro* at the indicated time points: 4 h, 8 h, 24 h, and 72 h. **(B)** The expression level of *SOCS1* in G-CSF-stimulated $CD3^+$ T cells at 4 h from 3 independent healthy donors. Error bars represent the mean ± SEM values from 3 independent experiments. One-way ANOVA, *P<0.05, **P < 0.01, ***P< 0.001. **(C)** *SOCS1* expression levels were determined by quantitative real-time RT-PCR upon G-CSF treatment *in vitro* for 72 h. The *SOCS1* expression level was normalized to that of the PBS control. Error bars represent the mean ± SEM values from 3 independent healthy donors, *P<0.05. **(D-E)** A representative CSF3R (CD114) expression level of $CD3^+$ T cells was determined by flow cytometry upon G-CSF treatment at 4 h *in vitro*. **(F-G)** Flow cytometric analysis of IL-2 secretion in

G-CSF-stimulated $CD3^+$ T cells at 72 h *in vitro*. **(H-I)** Flow cytometric analysis of T cell activation marker CD25 and CD69 in G-CSF-stimulated $CD3^+$ T cells at 4 h *in vitro*. **(J-K)** Flow cytometric analysis of phosphorylation level of STAT1, STAT3 and STAT3 in G-CSF-stimulated $CD3^+$ T cells at 4 h *in vitro*. Error bars represent the mean ± SEM values from 3-7 independent healthy donors, $^*P<0.05$.

Figure S7. Phenotype of SOCS1-overexpressing primary T cells.

(A) Strategy for FACS analysis in SOCS1-overexpressing T cells. **(B)** Flow cytometric analysis of TIGIT and Tim3 expression levels in SOCS1-overexpressing $CD3^+$ T cells. **(C)** Statistical analysis of the expression levels of the exhaustion markers 2B4, CD160, PD-1, TIGIT, and Tim-3 in SOCS1-overexpressing CD3+ T cells. Error bars represent the mean ± SEM values from 3 independent experiments from 3 healthy donors, $^*P<0.05$.

Figure S8. Cytokine secretion in SOCS1-overexpressing primary T cells.

(A-B) Flow cytometric analysis (A) and statistical results (B) of the IL-2, IFN-γ, and IL-17 secretion levels in the SOCS1-overexpressing $CD4^+$ T cells or $CD8^+$ T cells. Error bars represent the mean ± SEM values from 3 independent experiments from 3 healthy donors. **(C)** Flow cytometric analysis of the IL-4 and IL-10 secretion levels in the SOCS1-overexpressing $CD4^+$ T cells. Error bars represent the mean ± SEM values from 3 independent experiments from 3 healthy donors.

Figure S9. IL-10 secretion in SOCS1-inhibiting primary T cells.

(A) SOCS1 knockdown by siRNA in $CD3^+$ T cells from post-G healthy donors. The relative expression level of *SOCS1* was detected by quantitative real-time RT-PCR. Error bars represent the mean ± SEM values from 6 independent post-G healthy donors, $^{***}P<$ 0.001. **(B-C)** The IL-10 secretion level was detected by flow cytometry in $CD4^+$ T cells (B) or $CD8^+$ T cells (C) after SOCS1 was knocked down. Error bars represent the mean ± SEM values from 6 independent post-G healthy donors. **(D)** The IL-10 secretion level was detected by ELISAs in $CD3^+$ T cells after

SOCS1 was knocked down. Error bars represent the mean ± SEM values from 6 independent post-G healthy donors.

Figure S10. Phenotype of *Socs1* cKO Mice.

(A) Representative appearance of a 4-week-old cKO mouse. **(B)** Representative macroscopic view of the spleen from the *Socs1*$^{fl/fl}$ (WT) mouse and its age (8 weeks old)- and sex-matched cKO counterpart. **(C)** Representative flow cytometry results and percentages show the CD62L and CD44 expression levels on CD8$^+$ T cells from the spleens of WT or cKO mice. Naïve T cells: CD62L$^+$CD44$^-$; Central memory T cells (T_{CM}): CD62L$^+$CD44$^+$; Effector memory T cells (T_{EM}): CD62L$^-$CD44$^+$. **(D)** Absolute number of CD3$^+$, CD4$^+$, CD8$^+$ T cells in spleen of WT or cKO mice. **(E)** Absolute number of naïve T cells, T_{CM} and $_{TEM}$ from CD4$^+$ T cells in spleen of WT or cKO mice. **(F)** Absolute number of naïve T cells, T_{CM} and T_{EM} from CD8$^+$ T cells in spleen of WT or cKO mice. **(G)** Representative flow cytometry results show the Treg cell subsets in CD4$^+$ T cells from the spleens of WT or cKO mice. **(H)** Foxp3 expression level in Treg cells from the spleens of WT or cKO mice. **(I)** Representative flow cytometry results of the proliferation ability of Teff cells from WT mice after cocultured with Treg cells from WT or cKO mice respectively. The experiment was repeated at least 3 times, with 4-6 mice per group. Error bars represent the mean ± SEM, ***$P<0.001$, **$P<0.01$, *$P<0.05$.

Figure S11. GVHD model of *Socs1* cKO and WT Mice.

(A) Weight of the GVHD mice. 10 mice per group. **(B)** Hematoxylin and eosin stained sections of lung, liver, small intestine and large intestine from GVHD mice. Scale bars = 50 µm.

Figure S12. Phenotype of G-CSF administrated *Socs1* cKO Mice.

(A) Treg cell subsets in CD4$^+$ T cells from the spleens of WT or cKO mice treated with PBS or G-CSF respectively. **(B)** Foxp3 expression level in Treg cells from the spleens of WT or cKO mice treated with PBS or G-CSF respectively. **(C)** CD25$^+$ T

cell subsets in $CD4^+$ T cells from the spleens of WT or cKO mice treated with PBS or G-CSF respectively. The experiment was repeated at least 3 times, with 4-6 mice per group. Error bars represent the mean ± SEM, **P*<0.05.

Table S1. Characteristics of allo-HSCT patients with aGVHD and without aGVHD

**Characteristics**	**aGVHD (N=8)**	**Non-aGVHD (N=6)**	***P*-Value***
Days post-HSCT	43(13-86)	25(18-57)	0.228
Blood cell count			
Median WBC (×10^9/L) (range)	4.32 (1.98-19.27)	5.3(1.60-8.33)	0.66
Median ANC (×10^9/L) (range)	4.04(1.17-17.40)	4.04(1.13-7.69)	0.66
Median Hb (g/L) (range)	89(61-96)	86(64-109)	0.66
Median PLT (×10^9/L) (range)	81(26-206)	41(19-80)	0.18
Age at HSCT (years, median, range)	32(15-63)	29(11-55)	0.41
Gender (male/female)	6/2	2/4	0.28
Underlying disease			1.00
AML	5	3	
ALL	3	3	
MDS	0	0	
Status at HSCT			0.58
Standard-risk	6	3	
High-risk	2	3	
Source of stem cell			1.00
PB	8	6	
Transplanted total nucleated cell dose (×10^8/ kg, median, range)	8.53 (7.01-13.97)	10.09(7.84-13.90)	0.57
Transplanted $CD34^+$ cell dose (×10^6/ kg, median, range)	3.29(1.52-6.55)	3.20(1.66-4.67)	0.57
Donor match			1.00
HLA-identical sibling donor	2	2	
HLA-partially matched related donor	6	4	
Donor gender			0.58
Female	3	1	
male	5	5	
Donor-recipient pair			0.47
Female to male	2	0	
Others	6	6	
Blood type matching			0.25
Match	7	3	
Mismatch	1	3	
Pre-HSCT cycles of chemotherapy	4 (2-8)	5.5(3-7)	0.36
Conditioning			1.00
BU/CY	2	2	
BU/CY+ATG	6	4	
History of CMV reactivation	4	2	1.00
Onset of CMV reactivation (days, median, range)	11(0-49)	0(0-34)	0.49

*****Continuous variables were compared using the Mann-Whitney U test; categorical variables were compared using Fisher's exact test. $P<0.05$ was considered significant.

Abbreviations: allo-HSCT indicates allogeneic hematopoietic stem cell transplantation; aGVHD, acute graft-versus-host disease; PB, peripheral blood; WBC, white blood cell; ANC, absolute neutrophil cell; Hb, hemoglobin; PLT, platelet; AML, acute myelogenous leukemia; ALL, acute lymphocytic leukemia; HLA, human leukocyte antigen; ATG, antithymocyte globulin; CMV, cytomegalovirus.

Table S2. Characteristics of donors and patients with aGVHD and without aGVHD.

Characteristics	aGVHD group (N=9)	Non-GVHD group (N=9)	*P*-Value*
Donor gender			0.62
Male	7	5	
Female	2	4	
Donor age, median(range)	35.5(16-54)	39(16-63)	0.436
Patient gender			0.62
Male	5	7	
Female	4	2	
Patient age, median(range)	25(7-62)	29(6-56)	0.136
Underlying disease			0.70
AML	2	1	
ALL	6	6	
Others	1	2	
Source of stem cell			1.00
BM and PB	9	9	
Donor match			1.00
HLA-matched sibling donor	1	1	
Haploidentical related donor	8	8	
Transplanted total nucleated cell dose($\times 10^8$/ kg)	11.27(8.04-12.34)	9.68(7.39-13.88)	0.34
Transplanted BM nucleated cell dose($\times 10^8$/ kg)	4.47(3.00-6.22)	2.36(2.00-3.49)	0.001
Transplanted PB nucleated cell dose($\times 10^8$/ kg)	6.61(2.31-9.28)	6.37(5.00-10.39)	1.00
Transplanted $CD34^+$ cell dose($\times 10^6$/ kg)	2.45(1.47-5.88)	3.15(1.04-6.29)	0.796
Transplanted $CD3^+$ cell dose($\times 10^8$/ kg)	2.35 (0.14-6.08)	3.40(0.37-6.52)	0.55
Transplanted $CD4^+$ cell dose($\times 10^8$/ kg)	2.12(0.11-3.84)	2.00(0.34-7.36)	0.73
Transplanted $CD8^+$ cell dose($\times 10^8$/ kg)	0.30(0.09-0.75)	0.26(0.12-0.52)	0.67
Transplanted $CD4^-CD8^-$ cell dose($\times 10^8$/ kg)	0.16(0.09-0.59)	0.16(0.05-0.39)	0.93
Transplanted $CD14^+$ cell dose($\times 10^8$/ kg)	1.57(0.11-2.51)	1.75(0.29-3.81)	0.49
Conditioning			1.00
BU/CY	1	1	
BU/CY+ATG	8	8	

*Continuous variables were compared using the Mann-Whitney U test; categorical variables were compared using Fisher's exact test. P<0.05 was considered significant.

Abbreviations: BM, bone marrow; PB, peripheral blood; AML, acute myelogenous leukemia; ALL, acute lymphocytic leukemia; ATG, antithymocyte globulin; aGVHD, acute graft-versus-host disease.

Table S3. Antibody information.

Manufacturer	Name	Format	Clone	Cat.	Species
BD Pharmingen	CD4	Percp-Cy5.5	RPA-T4	560650	Human
BD Pharmingen	CD4	APC-H7	RPA-T4	560158	Human
BD Horizon	CD8	APC-R700	RPA-T8	565165	Human
BD Horizon	CD8	V500	RPA-T8	560774	Human
BD Pharmingen	PD-1	PE-Cy7	EH12.1	561272	Human
eBioscience	TIM-3	APC	F38-2E2	17-3109-42	Human
Biolegend	TIGIT	BV605	A15153G	372712	Human
Biolegend	2B4	Alexa-Fluor®700	C1.7	329526	Human
BD Pharmingen	CD160	PE	BY55	562118	Human
BD Pharmingen	Ki-67	PE	B56	556027	Human
BD Horizon	IL-2	V450	5344.111	560867	Human
Biolegend	IFN-γ	BV510	4S.B3	502544	Human
BD Pharmingen	IL-17	PE	N49-653	560487	Human
BD Pharmingen	IL-4	APC	8D4-8	560671	Human
BD Pharmingen	IL-10	PE	JES3-9D7	554498	Human
Biolegend	P-STAT1	Alexa Fluor® 647	A17012A	666409	Human
Biolegend	P-STAT3	Alexa-Fluor®647	13A3-1	651008	Human
Biolegend	P-STAT5	PE	A17016B	936903	Human
BD Pharmingen	CD3e	PerCP	145-2C11	553067	Mouse
BD Pharmingen	CD4	APC-H7	GK1.5	560181	Mouse
Biolegend	CD4	PE	GK1.5	100408	Mouse
Biolegend	CD8	FITC	53-6.7	100706	Mouse
Biolegend	CD25	APC	PC61	102012	Mouse
Biolegend	CD44	PE-Cy7	IM7	103030	Mouse
Biolegend	CD62L	APC	MEL-14	104411	Mouse
BD Pharmingen	IFN-γ	PE	XMG1.2	554412	Mouse
Biolegend	CD8	Alexa-Fluor®700	53-6.7	100730	Mouse
BD Horizon	CFSE			565082	Mouse
Abcam	Histone H3		polyclone	ab4729	Human
CST	P-STAT3		D3A7	9145	Human/Mouse
CST	STAT3		79D7	4904	Human/Mouse
CST	SOCS1		A156	3950	Human/Mouse
CST	β-Actin		8H10D10	3700	Human/Mouse
abclonal	CTCF		polyclone	A1133	Human

Reference

[1] N. Servant, B. R. Lajoie, E. P. Nora, L. Giorgetti, C. J. Chen, E. Heard, J. Dekker, E. Barillot, *Bioinformatics* **2012**, *28*, 2843, https://doi.org/10.1093/bioinformatics/bts521.

[2] A. R. Barutcu, B. R. Lajoie, R. P. McCord, C. E. Tye, D. Hong, T. L. Messier, G. Browne, A. J. van Wijnen, J. B. Lian, J. L. Stein, J. Dekker, A. N. Imbalzano, G. S. Stein, *Genome biology* **2015**, *16* (1), 214, https://doi.org/10.1186/s13059-015-0768-0.

[3] E. Crane, Q. Bian, R. P. McCord, B. R. Lajoie, B. S. Wheeler, E. J. Ralston, S. Uzawa, J. Dekker, B. J. Meyer, *Nature* **2015**, *523* (7559), 240, https://doi.org/10.1038/nature14450.

[4] P. C. Taberlay, J. Achinger-Kawecka, A. T. L. L. Lun, A. Fabian, D. C. Bauer, G. K. Smyth, C. Stirzaker, S. I. O. Donoghue, F. A. Buske, K. Sabir, C. M. Gould, E. Zotenko, S. A. Bert, K. A. Giles, D. C. Bauer, G. K. Smyth, C. Stirzaker, S. I. O'Donoghue, S. Clark, *Genome research* **2016**, *April 8*, gr.201517.115, https://doi.org/10.1101/gr.201517.115.

[5] A. R. Quinlan, I. M. Hall, *Bioinformatics* **2010**, *26* (6), 841, https://doi.org/10.1093/bioinformatics/btq033.

[6] D. W. Huang, B. T. Sherman, R. A. Lempicki, *Nucleic acids research* **2009**, *37* (1), 1, https://doi.org/10.1093/nar/gkn923.

[7] H. S. Kaya-Okur, S. J. Wu, C. A. Codomo, E. S. Pledger, T. D. Bryson, J. G. Henikoff, K. Ahmad, S. Henikoff, *Nat Commun* **2019**, *10* (1), 1930, https://doi.org/10.1038/s41467-019-09982-5.
