# Supplementary material for: Multiomics Analysis Identifies SOCS1 as Restraining T Cell Activation and Preventing Graft‐Versus‐Host Disease
Source: Adv Sci (Weinh). 2022 May 18;9(21):2200978. doi: 10.1002/advs.202200978 (PMC9313503; doi:10.1002/advs.202200978)
Supplement: Supplementary file 2 — Supporting Information [file ADVS-9-2200978-s001.pptx]

## Slide 1
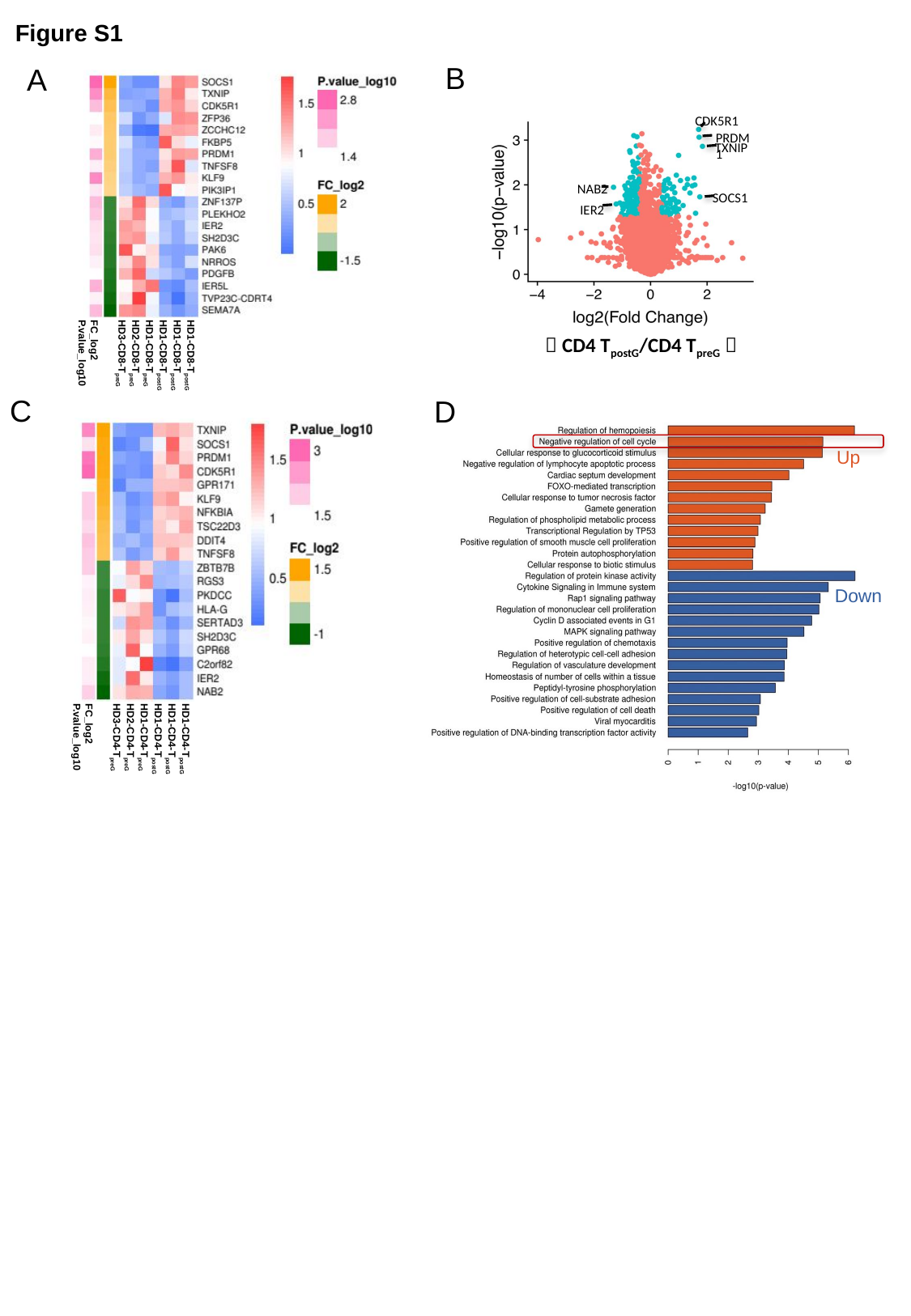

Figure S1
B
A
HD1-CD8-TpostG
HD1-CD8-TpostG
HD1-CD8-TpostG
HD1-CD8-TpreG
HD2-CD8-TpreG
HD3-CD8-TpreG
FC_log2
P.value_log10
CDK5R1
PRDM1
TXNIP
NAB2
SOCS1
IER2
（CD4 TpostG/CD4 TpreG）
C
D
HD1-CD4-TpostG
HD1-CD4-TpostG
HD1-CD4-TpostG
HD1-CD4-TpreG
HD2-CD4-TpreG
HD3-CD4-TpreG
FC_log2
P.value_log10
Up
Down

## Slide 2
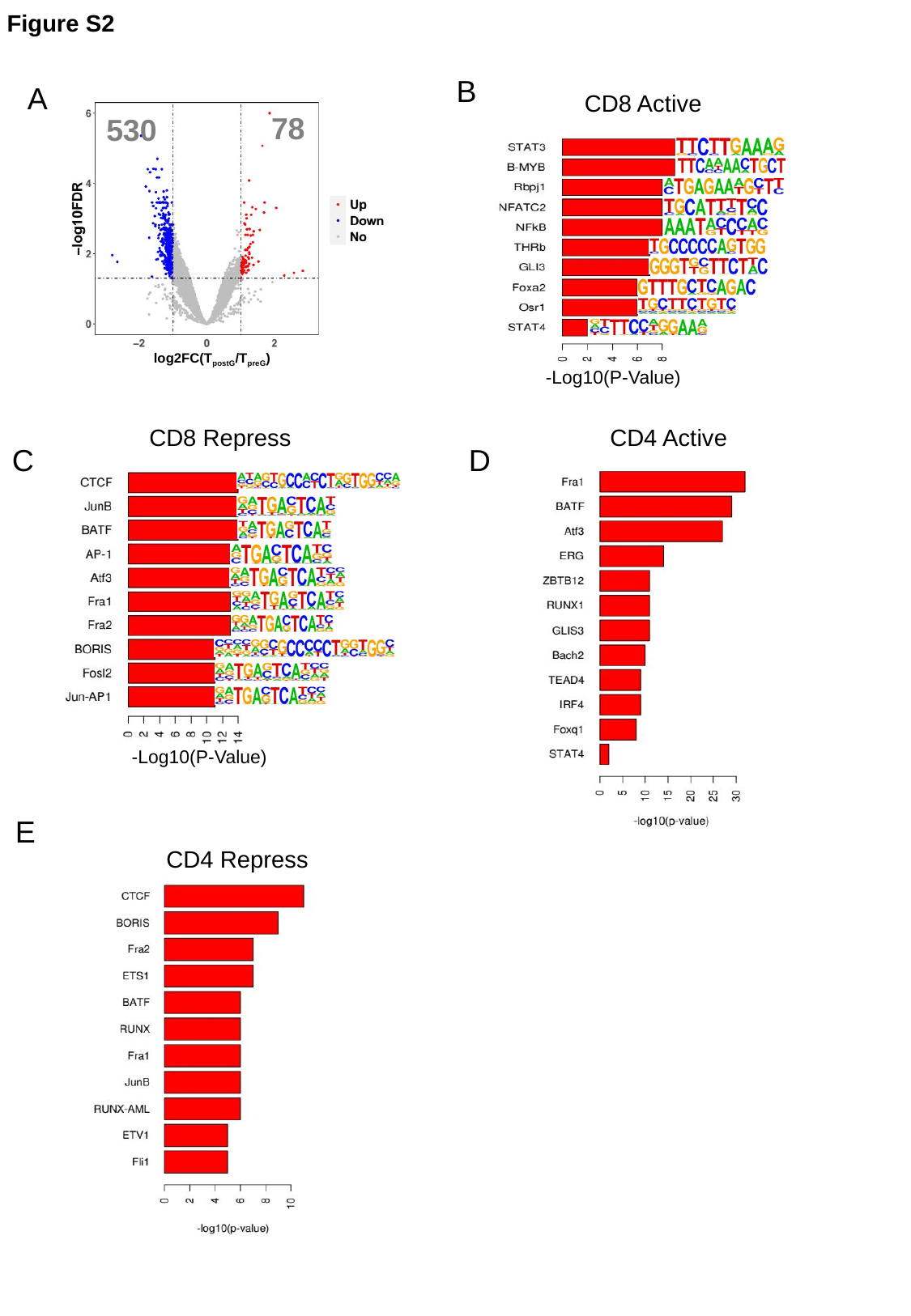

Figure S2
B
A
CD8 Active
-Log10(P-Value)
log2FC(TpostG/TpreG)
78
530
CD4 Active
CD8 Repress
-Log10(P-Value)
C
D
E
CD4 Repress

## Slide 3
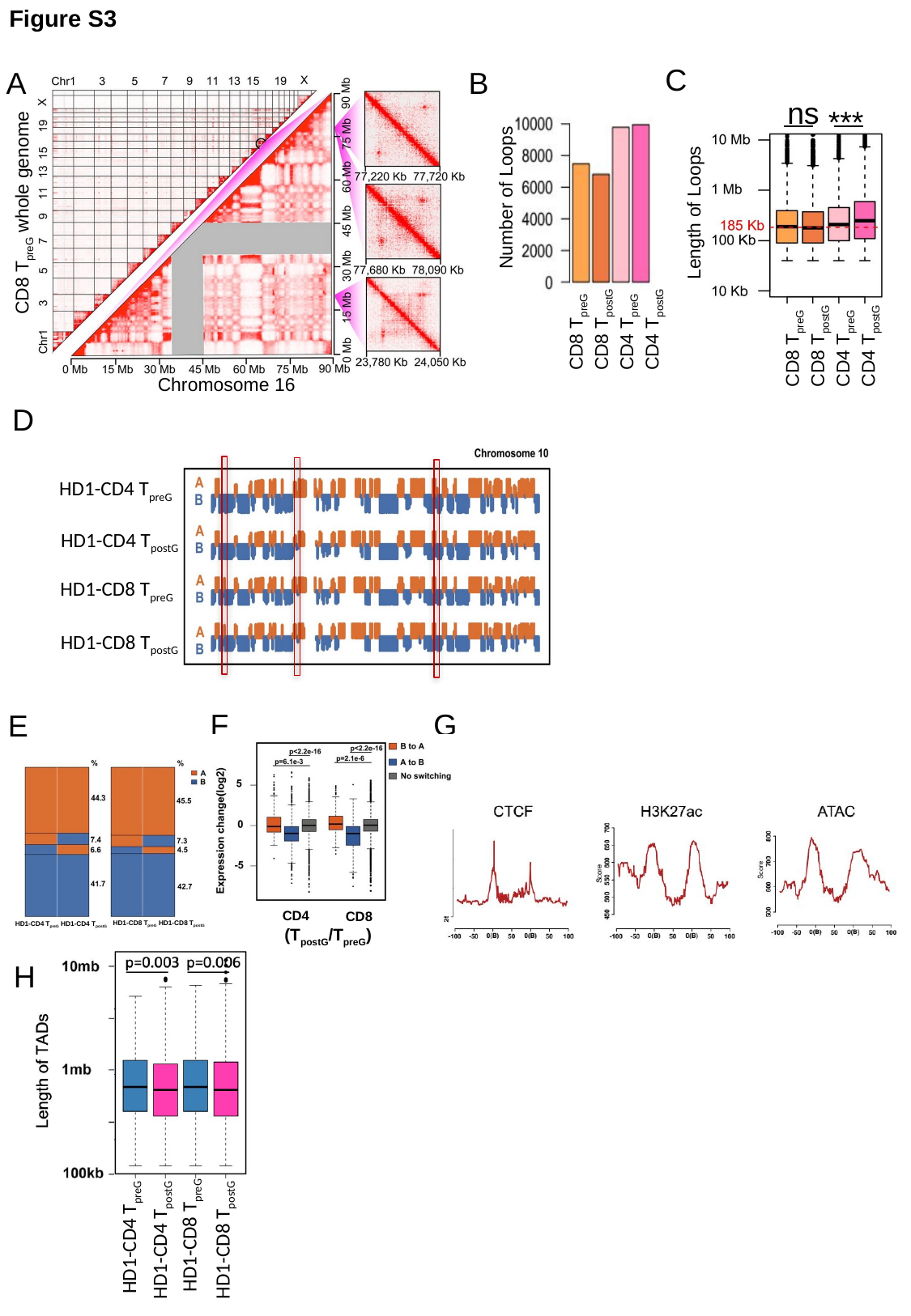

Figure S3
C
A
B
CD8 TpreG whole genome
Chromosome 16
ns
***
Length of Loops
CD8 TpreG
CD8 TpostG
CD4 TpreG
CD4 TpostG
Number of Loops
CD8 TpreG
CD8 TpostG
CD4 TpreG
CD4 TpostG
D
HD1-CD4 TpostG
HD1-CD8 TpostG
HD1-CD8 TpreG
HD1-CD4 TpreG
G
F
E
CD4
CD8
(TpostG/TpreG)
HD1-CD8 TpreG HD1-CD8 TpostG
HD1-CD4 TpreG HD1-CD4 TpostG
HD1-CD8 TpreG
HD1-CD4 TpreG
HD1-CD4 TpostG
HD1-CD8 TpostG
H

## Slide 4
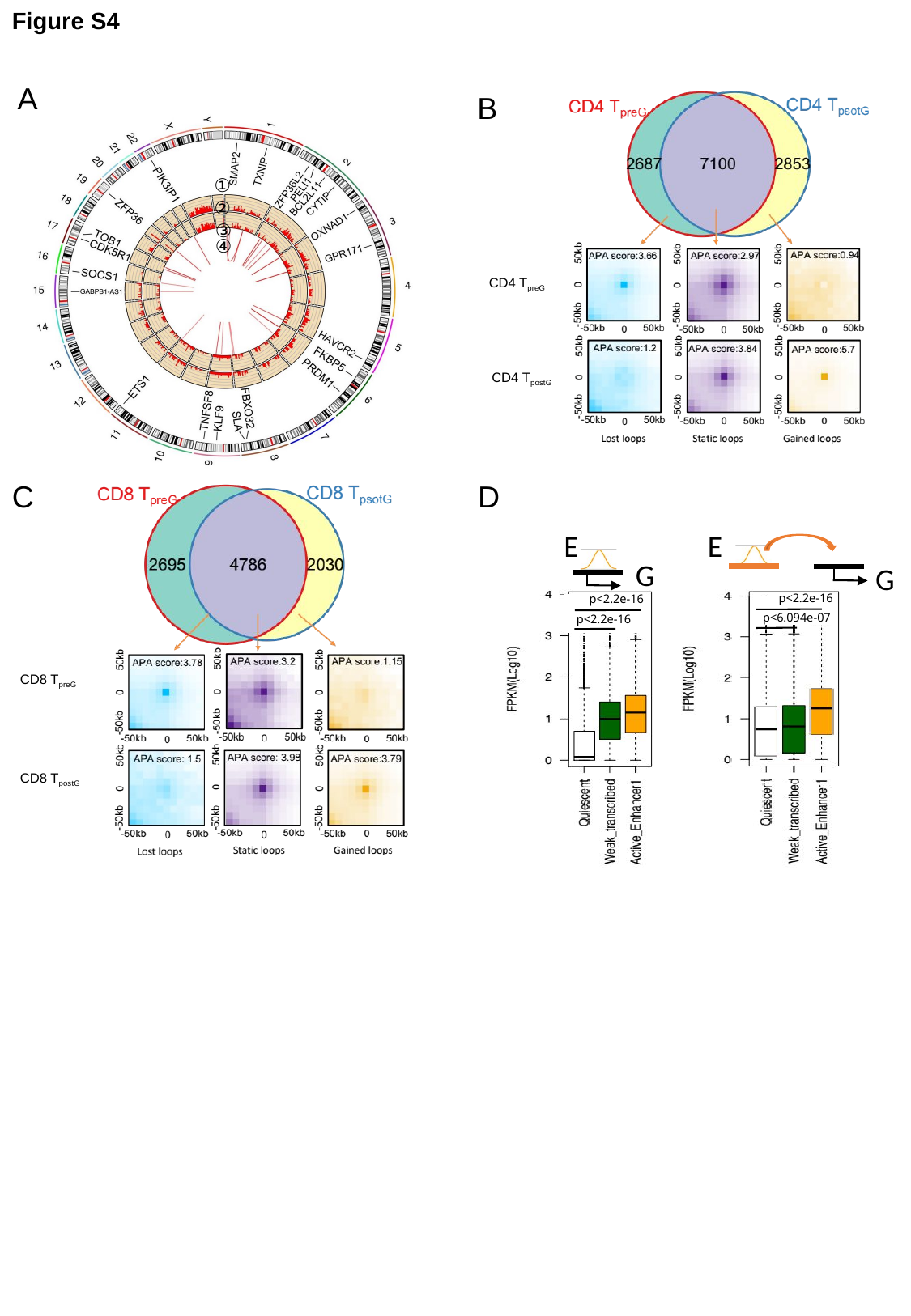

Figure S4
A
B
①
②
③
④
CD4 TpreG
CD4 TpostG
D
C
E
E
G
G
p<2.2e-16
p<6.094e-07
p<2.2e-16
p<2.2e-16
CD8 TpreG
CD8 TpostG

## Slide 5
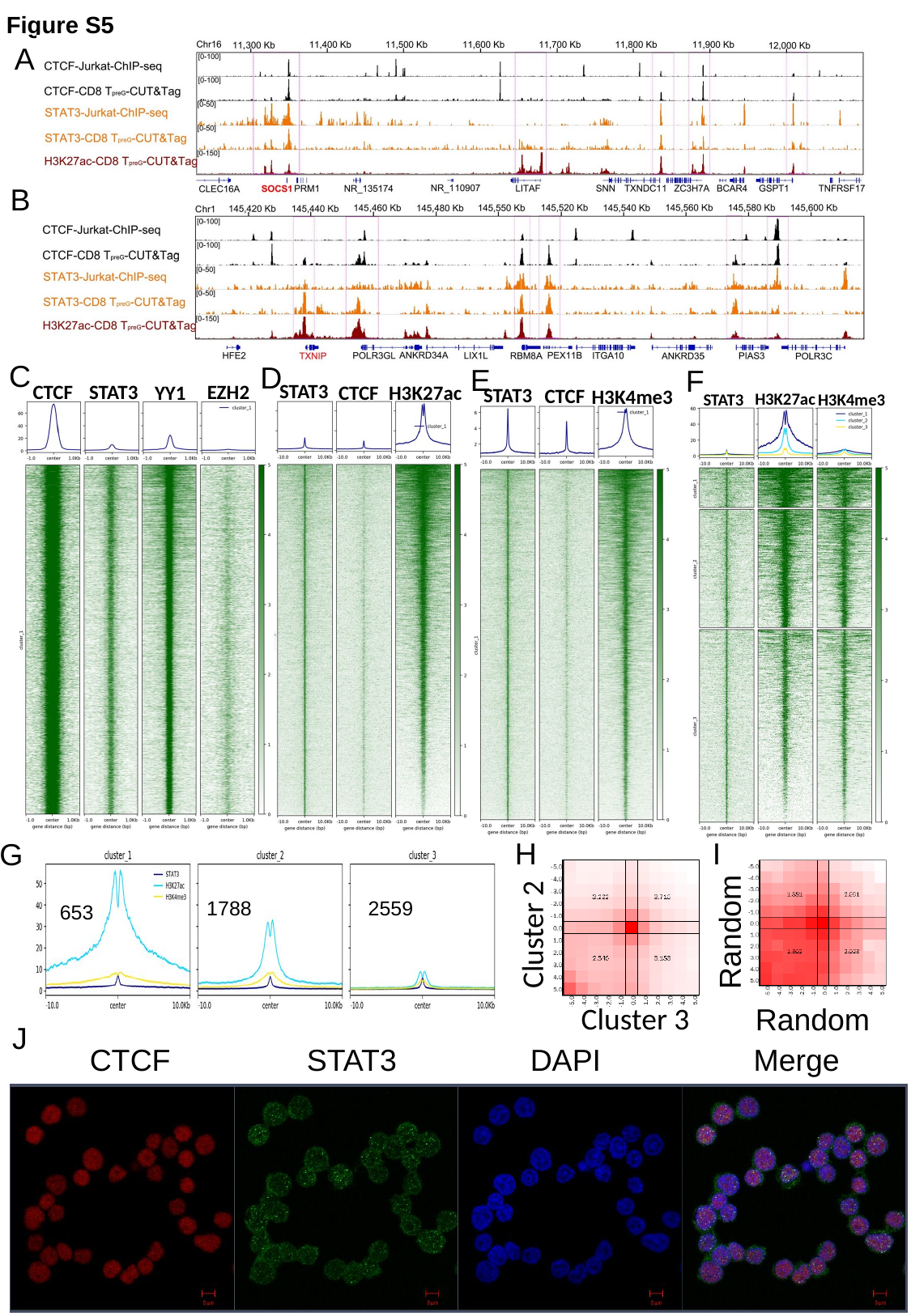

Figure S5
A
B
C
D
E
F
STAT3
H3K27ac
CTCF
STAT3
CTCF
YY1
EZH2
STAT3
CTCF
H3K4me3
H3K27ac
H3K4me3
STAT3
I
H
G
Cluster 2
Cluster 3
Random
Random
2559
1788
653
J
CTCF
STAT3
DAPI
Merge

## Slide 6
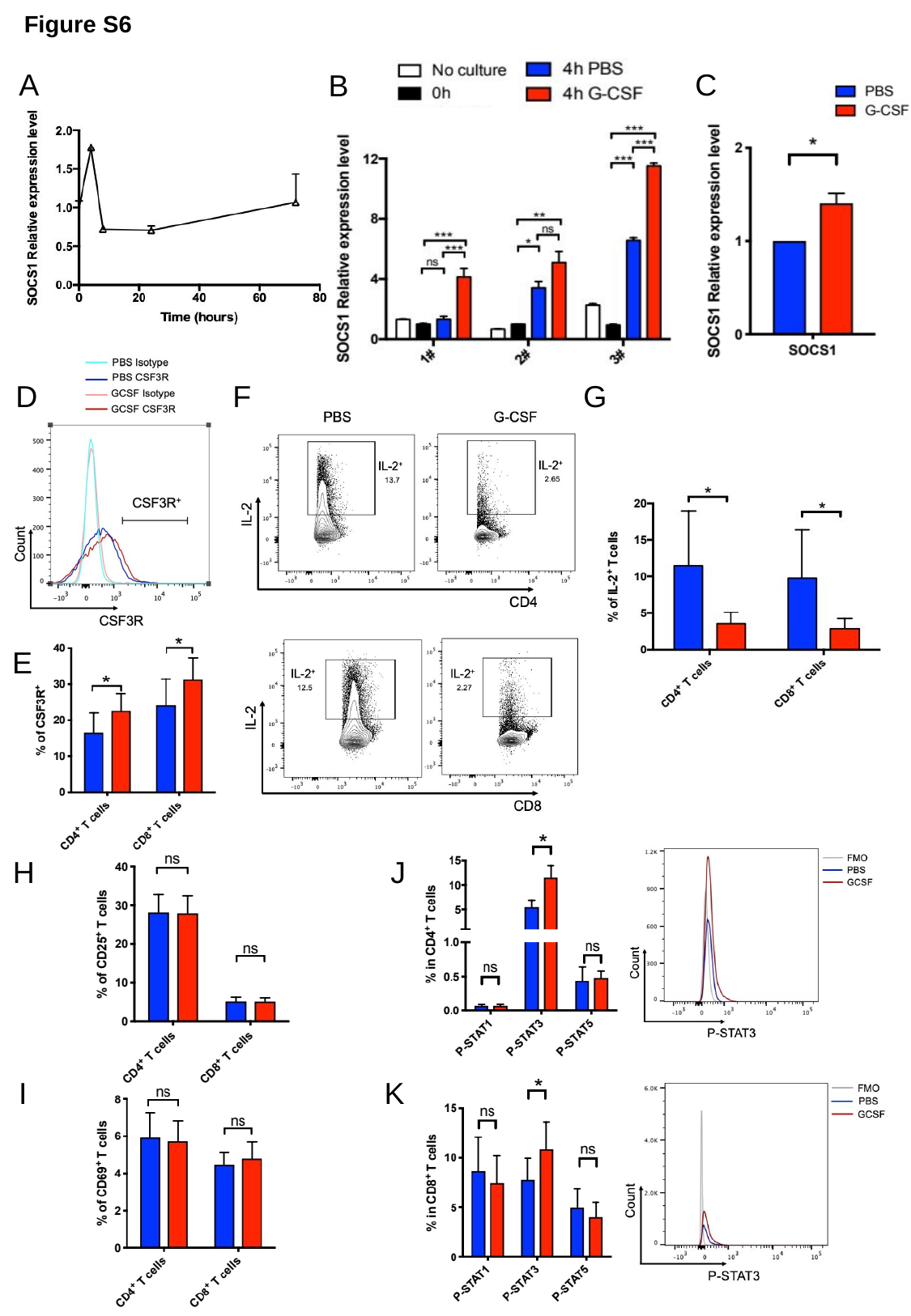

Figure S6
A
C
B
D
F
G
E
H
J
I
K

## Slide 7
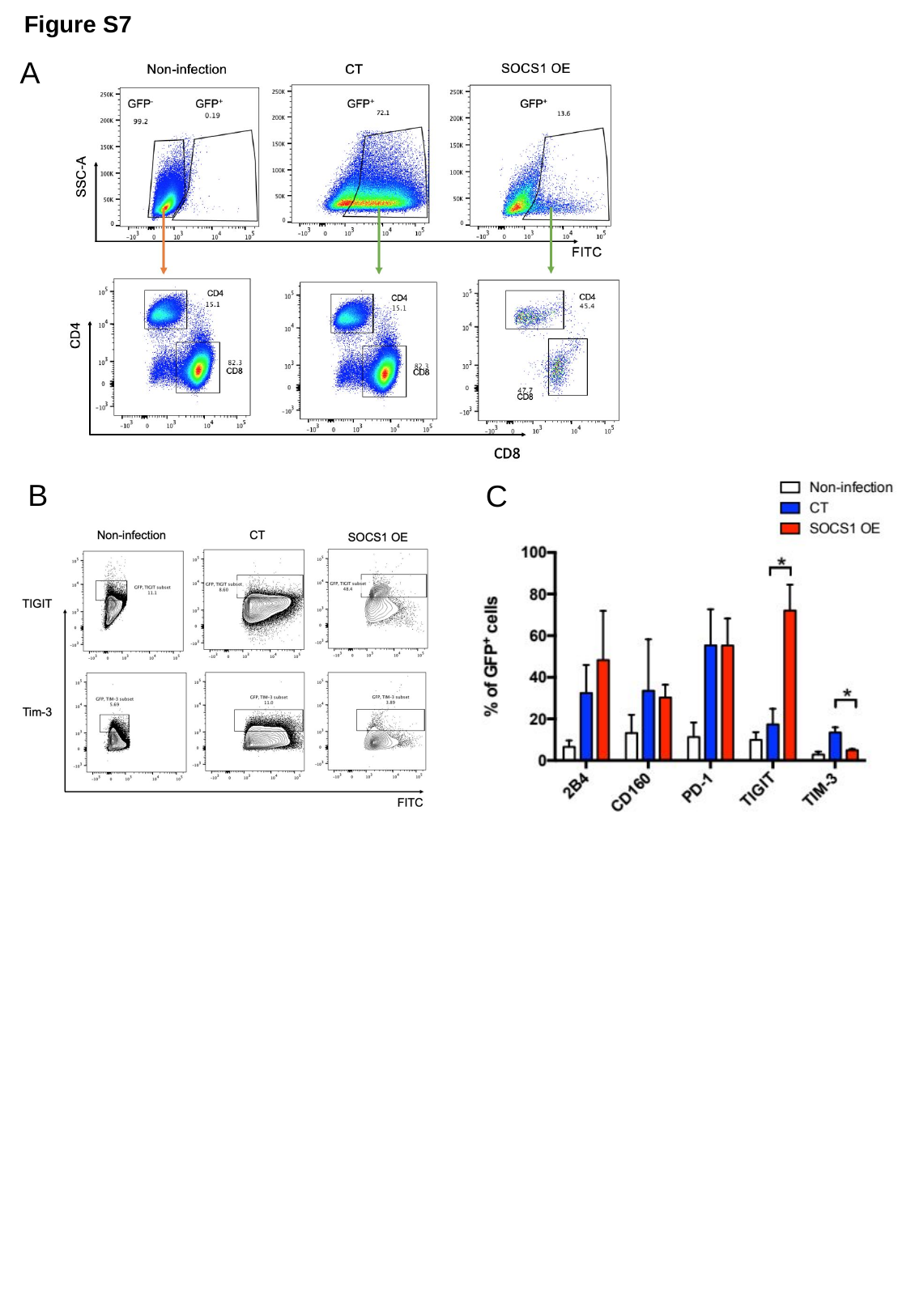

Figure S7
A
B
C

## Slide 8
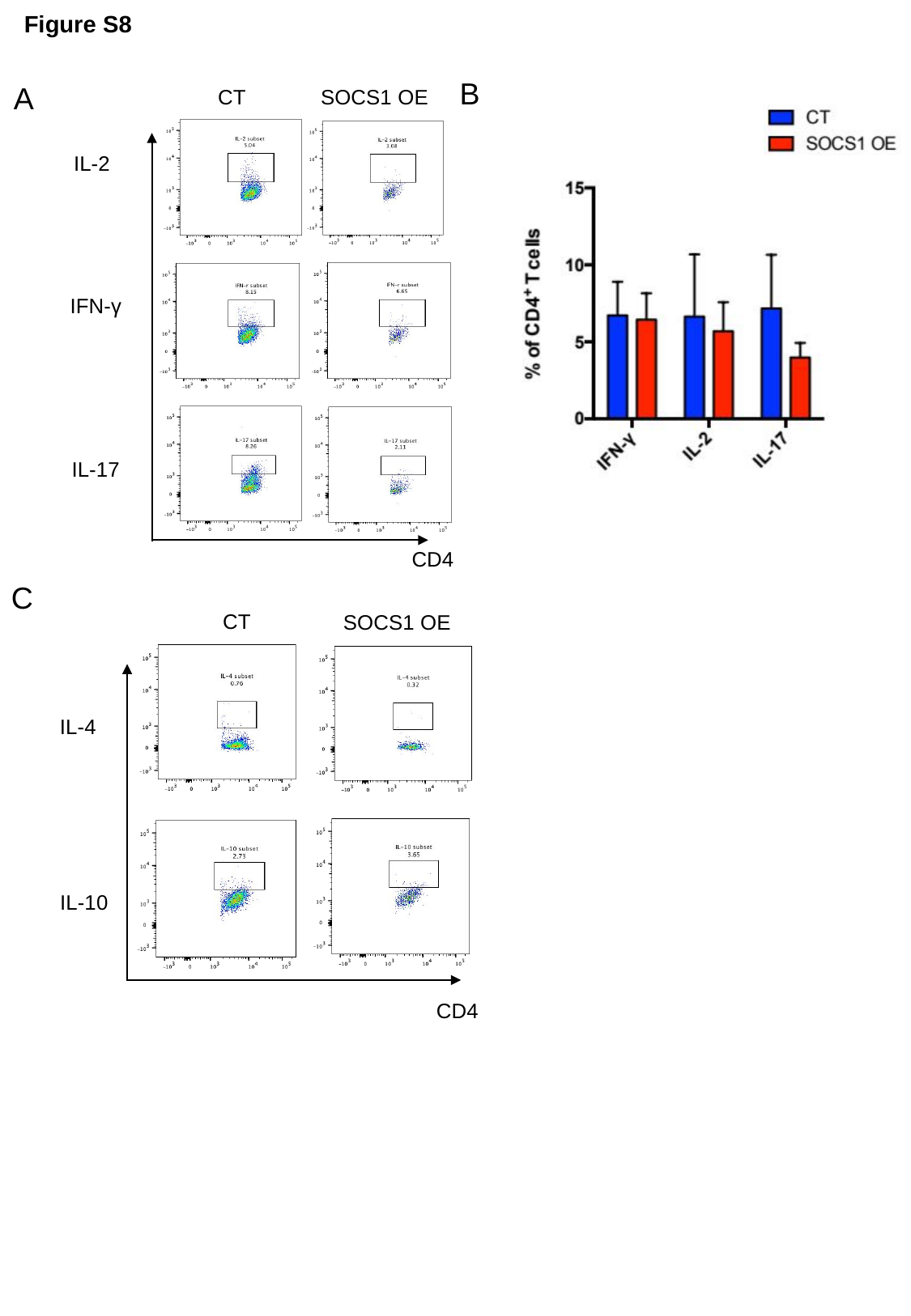

Figure S8
B
A
CT
SOCS1 OE
IL-2
IFN-γ
IL-17
CD4
C
CT
SOCS1 OE
IL-4
IL-10
CD4

## Slide 9
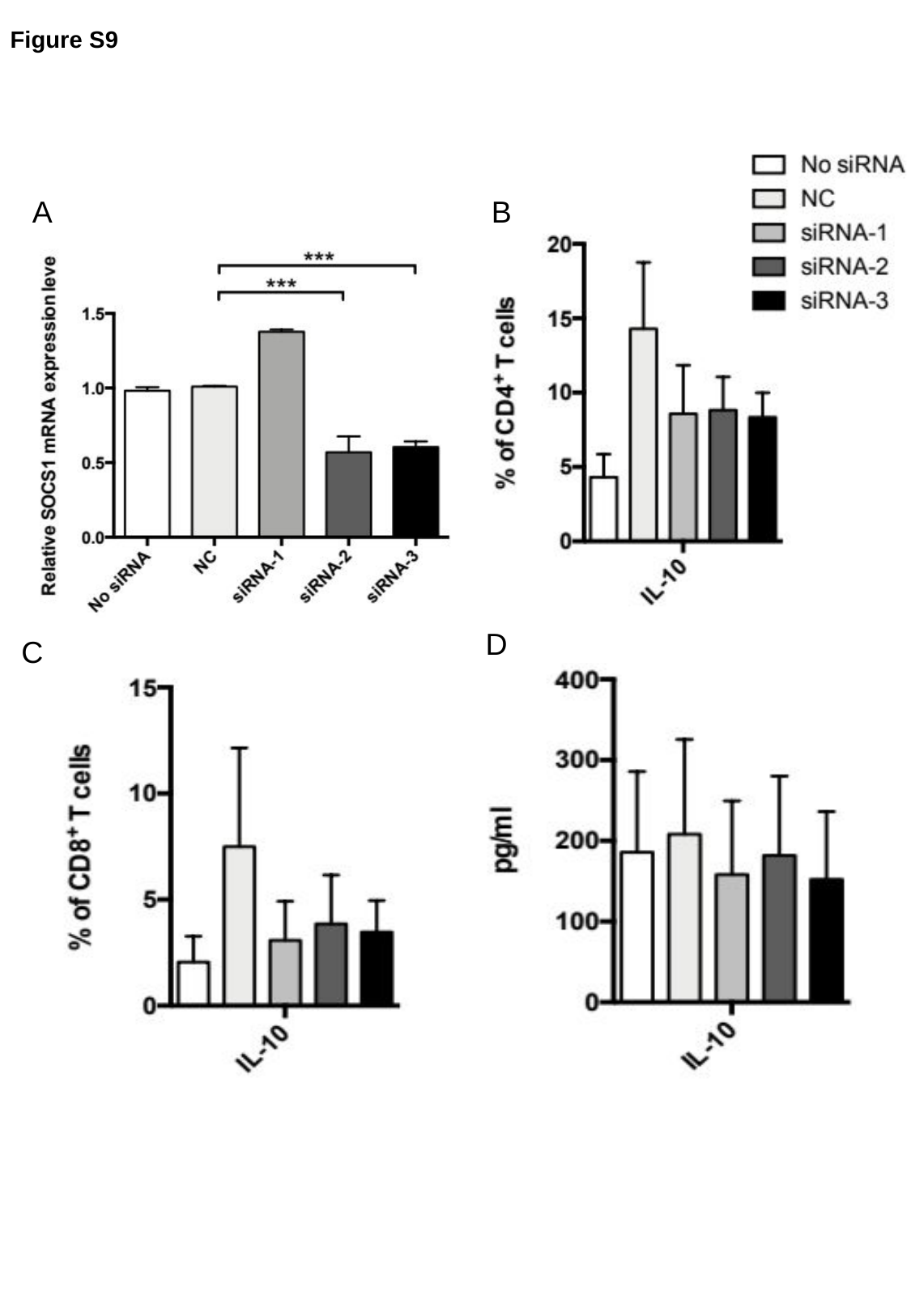

Figure S9
A
B
D
C

## Slide 10
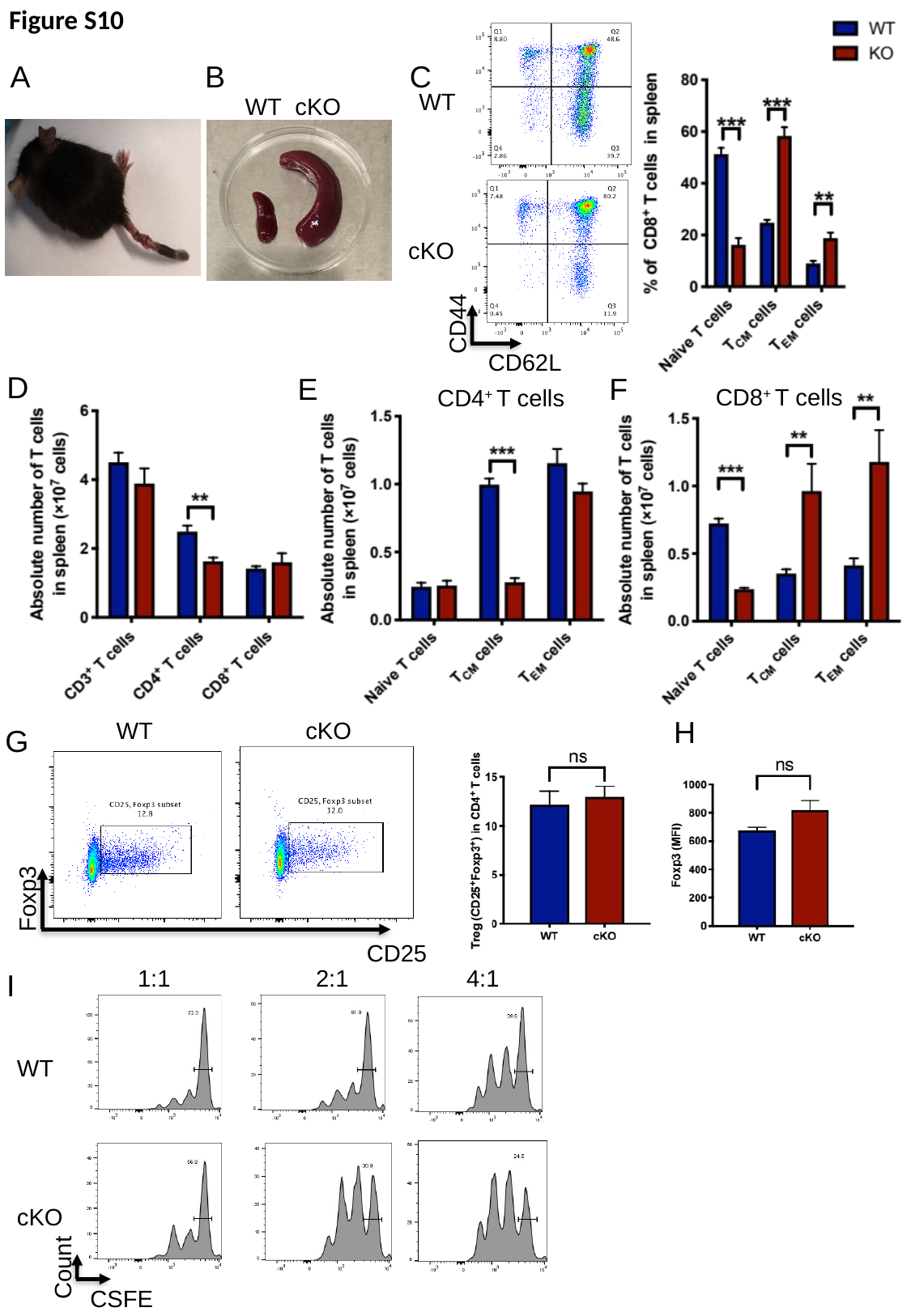

Figure S10
WT
cKO
CD44
CD62L
C
A
B
WT cKO
D
F
E
CD8+ T cells
CD4+ T cells
H
WT
cKO
Foxp3
CD25
G
4:1
1:1
2:1
WT
cKO
Count
CSFE
I

## Slide 11
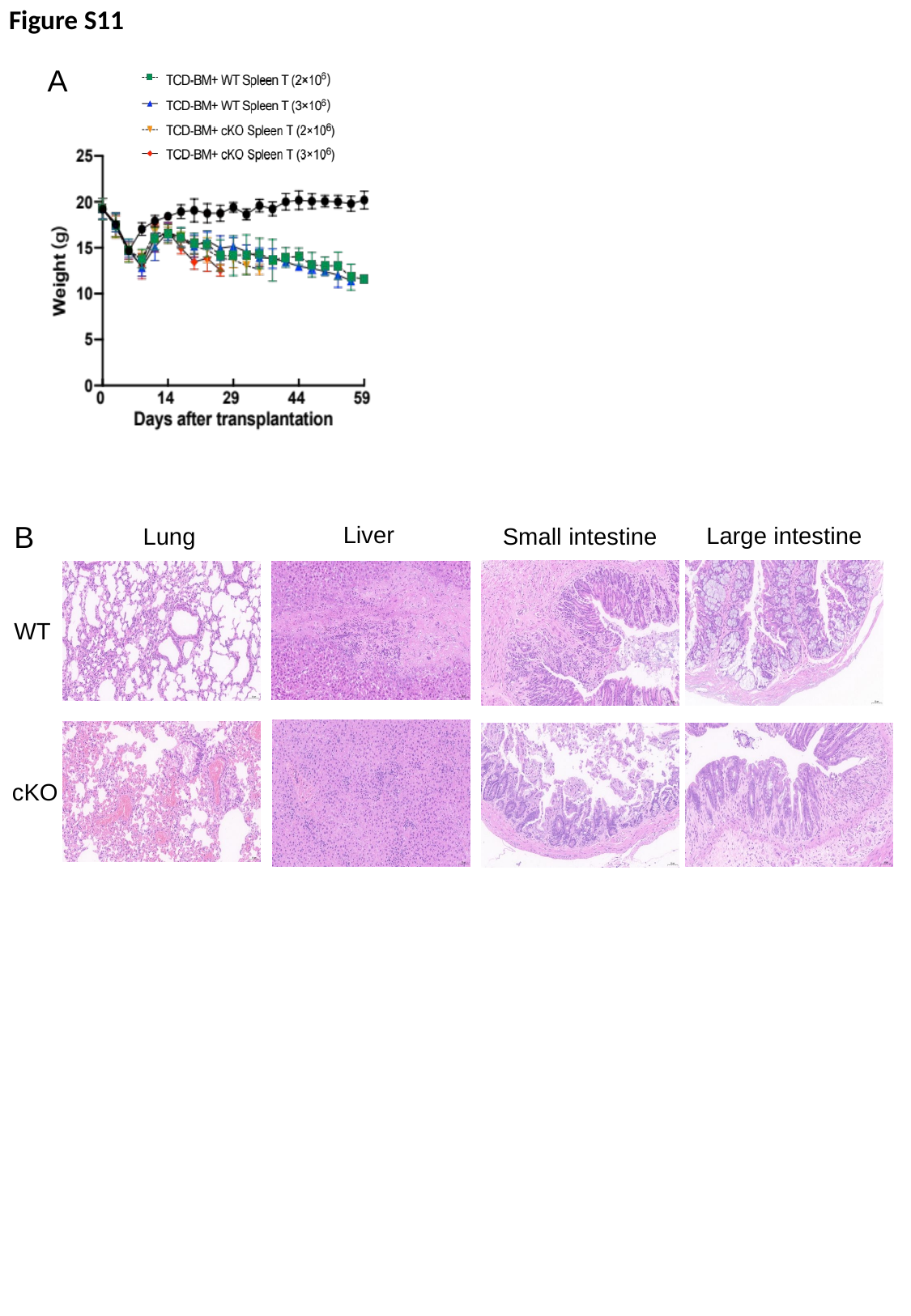

Figure S11
A
B
Liver
Large intestine
Lung
Small intestine
WT
cKO

## Slide 12
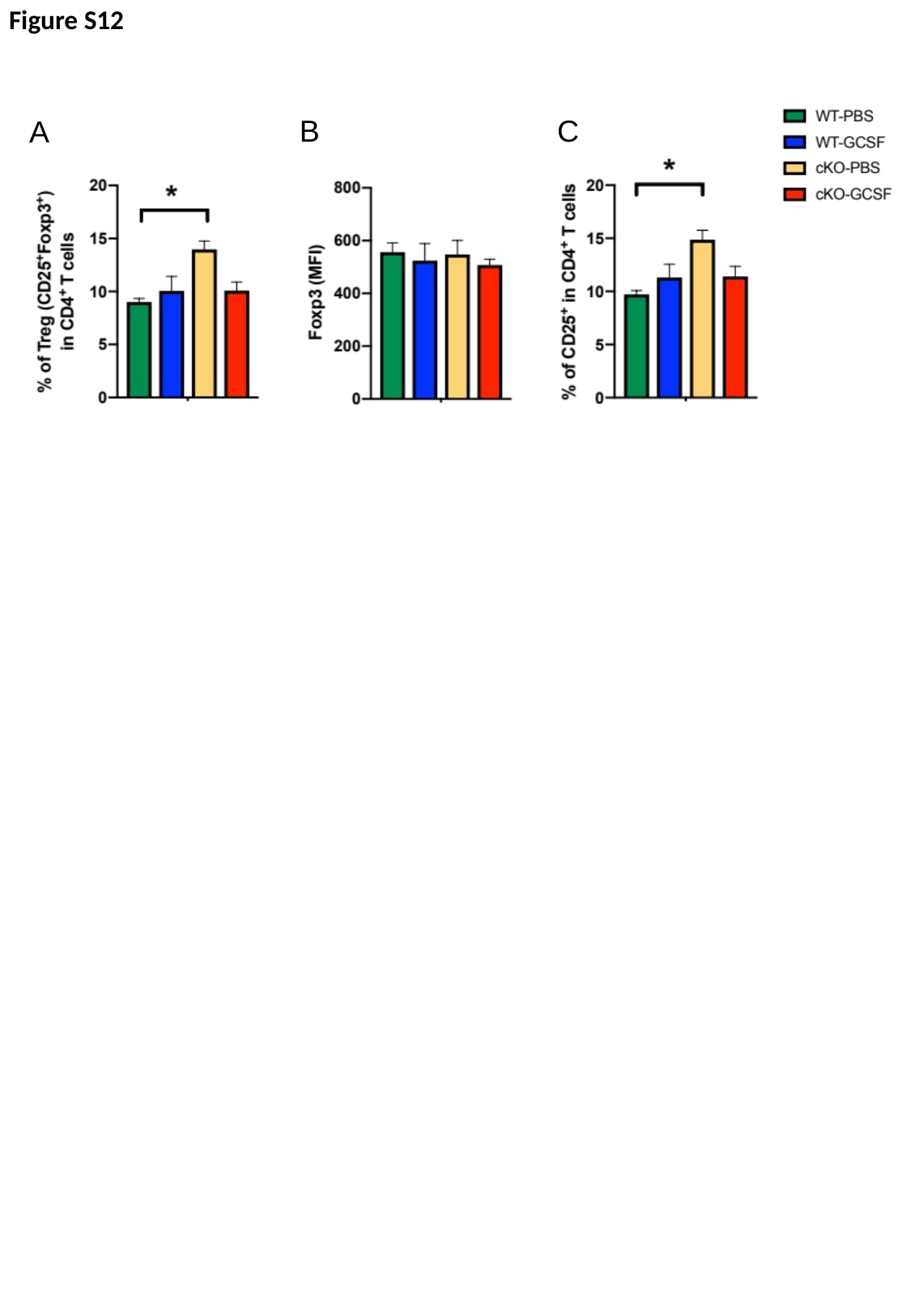

Figure S12
B
C
A
